# Environmental inequality in the neighborhood networks of urban mobility in US cities

**DOI:** 10.1073/pnas.2117776119

**Published:** 2022-04-21

**Authors:** Noli Brazil

**Affiliations:** ^a^Department of Human Ecology, University of California, Davis, CA 95616

**Keywords:** neighborhood, environmental justice, air quality, urban mobility, network

## Abstract

Research has made clear that neighborhoods impact the health and well-being of their residents. A related strand of research shows that neighborhood disadvantage is geographically clustered. Because the neighborhoods of low-income and minority populations tend to be more disadvantaged, neighborhood conditions help explain racial and socioeconomic inequalities. These strands of research restrict processes of neighborhood influence to operate only within and between geographically contiguous neighbors. However, we are underestimating the role of neighborhood conditions in explaining inequality if disadvantage extends beyond the residential and extralocal environments into a network of neighborhoods spanning the urban landscape based on where residents move within a city. I use anonymized mobile phone data to measure exposure to air pollution among residents of poor and minority neighborhoods in 88 of the most populous US cities. I find that residents from minority and poor neighborhoods travel to neighborhoods that have greater air pollution levels than the neighborhoods that residents from White and nonpoor neighborhoods visit. Hispanic neighborhoods exhibit the greatest overall pollution burden, Black/White and Asian/White disparities are greatest at the network than residential scale, and the socioeconomic advantage of lower risk exposure is highest for residents from White neighborhoods. These inequalities are notable given recent declines in segregation and air pollution levels in American cities.

## Significance

Exposure to air pollution within one’s residential neighborhood has detrimental consequences on health and well-being. Yet, this effect may be mitigated or exacerbated because individuals spend much of their time outside of their residential neighborhood to travel to neighborhoods across a city for work, errands, and leisure. Using mobile phone data to track neighborhood mobility in large US cities, I find that residents from minority and poor neighborhoods travel to neighborhoods that have greater air pollution levels than the neighborhoods that residents from White and nonpoor neighborhoods visit. These results reveal that minority and poor residents face environmental inequalities at three geographic scales: the neighborhoods they live in, their bordering neighborhoods, and the neighborhoods they visit.

A long line of research from a wide set of disciplines using a variety of methodological approaches on different kinds of data has demonstrated the negative consequences of living in disadvantaged neighborhoods on health and well-being (1–3). While debate still exists as to which neighborhood mechanisms matter, a large number of epidemiological studies have produced convincing evidence that exposure to environmental toxins in the neighborhood negatively impacts a variety of health outcomes (4–6). Because the neighborhoods of low-income and minority populations tend to be considerably more disadvantaged than those of comparable higher-income and White populations, exposure to adverse neighborhood conditions helps explain socioeconomic and racial inequalities (7, 8). For example, studies have found that minority and poor neighborhoods tend to have higher air pollution levels than White and nonpoor neighborhoods, partially explaining inequalities in individual health outcomes, including elevated risk of premature mortality from cardiovascular diseases and lung cancer and decreased cognitive functioning (6, 9–12). Another strand of research shows that neighborhood disadvantage is spatially clustered, with minority neighborhoods, even if they are relatively advantaged, surrounded by disadvantaged neighborhoods, further isolating them from opportunity-rich areas (13). In other words, neighborhoods with large concentrations of poor, Black, or Hispanic residents not only contain greater environmental hazards but also are surrounded by higher levels of environmental risks in the “extralocal” setting.

These two strands of research restrict processes of spatial disadvantage to operate only within neighborhoods and between geographically contiguous neighborhoods, similar to an infection spreading from a localized point source. The implication is that space matters, but that influence is constrained by distance and that nearest neighbors matter most. However, if residents spend significant time outside of their neighborhoods and travel to neighborhoods beyond those that are geographically adjacent, we are misestimating their exposure (14–17). Individuals living in neighborhoods with high air pollution levels may also travel to other high air pollution neighborhoods, thus exacerbating their health risks, especially if activities in these neighborhoods are done primarily outdoors (e.g., spending time at a park). Here, residents of racial minority and poor neighborhoods confront spatial disadvantage at three ecological scales: their residential neighborhood, neighborhoods adjacent to their residential neighborhood, and the network of neighborhoods connected by the ways they move around the city for work, errands, and leisure.

Several research perspectives motivate this neighborhood network framework. Most prominent is the work on activity spaces, which encompass the spatial contexts in which individuals conduct their daily activities (18, 19). The activity space framework recognizes that obligations, tasks, and social engagements may draw people out of, and potentially far from, their residential context. Therefore, relevant social spaces often emerge through the dynamics of individuals’ movement between and among neighborhoods. The literature on social networks also provides theoretical motivation for studying neighborhood networks. Social network theory predicts diffusion occurring through social ties, which may occur between actors spatially distant from one another (20, 21). The perspective adopted in this study moves away from the individual-level nodes and ties that social network and activity space research emphasize toward population-level flows connecting neighborhoods. Spatial mobility flows, especially those that are geographically distant, are shaped not just by individual happenstance or geographic proximity, but also by institutional ties or meaning frameworks (22), social distance between areas (23), and meso-level processes such as segregation and gentrification (24, 25). As flows between neighborhoods persist, origin and destination become linked, especially if counterflows exist and residents maintain ties in both communities (17). These sorts of interactions give the network its form and feed back into the ways it affects both communities and individuals.

Studies using data on work commuting flows, geolocation records from social media platforms, and other forms of mobility have found that urban mobility connects communities both near and far (17, 26–29). However, only a few of these studies have examined race or class differences in mobility, finding that minority and poor neighborhoods are generally isolated from White and nonpoor areas (28–31). Furthermore, even fewer studies have examined exposure to other neighborhood conditions outside of racial and poverty composition and how this exposure is stratified by race and class (32–34). In other words, although we know that residents travel to distant neighborhoods and the socioeconomic and racial compositions of these neighborhoods follow patterns of social isolation and segregation, we know little else about their other ecological features. This study fills these gaps by using anonymized mobile phone data to measure exposure to environmental toxins among residents of poor and minority neighborhoods in 88 of the most populous US cities. The study does not model causal pathways, but instead sheds light on the racial and socioeconomic neighborhood disparities in exposure to pollution levels brought about by the day-to-day mobility of residents within a city. I focus on air pollution because unlike demographic and socioeconomic conditions such as poverty and racial composition individuals are more likely to be directly exposed to air pollution, even if they are in the neighborhood for a brief period. Furthermore, while the mechanisms underlying the connections between individual health and neighborhood racial and poverty composition are still debated, the pathways connecting health and neighborhood exposure to environmental toxins are better understood (3, 6, 10, 14).

## Materials and Methods

All data were collected at the census tract level for the 100 most populated cities in the United States based on 2018 estimates. As relatively permanent subdivisions of a county designed to be homogeneous with respect to general population characteristics, census tracts are the most common proxy for neighborhoods in social science research (17). Cities are used as the broader geographic container to align the findings with prior work on neighborhood structural connectedness (27, 28, 34). Recognizing that mobility nontrivially extends beyond city boundaries, I examine the mobility of city residents to neighborhoods within the city as well as those located across the city’s wider metropolitan statistical area (MSA). An MSA represents a core area containing a substantial population nucleus, together with adjacent communities having a high degree of economic and social integration with that core.

Cell phone location data rely on numerous smart phone apps and were aggregated by SafeGraph, a company that builds and maintains anonymized geospatial datasets for more than 40 million US smartphones. SafeGraph provides visit patterns to more than 6 million points of interest in the United States to study mobility patterns and foot traffic. The dataset contains information on the daily number of pings in a destination block group and the home block group locations of the pings. SafeGraph defines a person’s “home” to be the location where the mobile device is detected most at night (from 18:00 to 07:00) over a 6-wk period. Location is defined at the Geohash-7 level (153 × 153-m grid). Block groups with fewer than two cell phone devices are excluded from the data (i.e., there must be at least two visitors). The SafeGraph sample of mobile devices closely corresponds to US Census population counts by state (correlation of *r* = 0.977 between SafeGraph and Census counts across state) and county (*r* = 0.966). Similarly, strong correlations appear to exist between Census counts and the estimated racial/ethnic composition (*r* = 1.00), education group (*r* = 0.999), and income (*r* = 0.997) (35). The analysis includes pings from November 2018 to November 2019 aggregated up to the census tract level.

Air pollution data come from the Environmental Protection Agency’s (EPA’s) Environmental Justice Screening and Mapping Tool EJSCREEN. Following prior work employing an environmental justice framework, I measure air pollution exposure using particulate matter ($PM_{2.5}$) levels (36). $PM_{2.5}$ (μg/m^3^) describes fine inhalable particles, with diameters that are generally 2.5 *μ*m and smaller, and levels are calculated by the EPA using a fusion of modeled and monitored data collected from a nationwide network of monitoring sites. The most recent national data for average annual $PM_{2.5}$ (2017) were used.

I examined patterns of $PM_{2.5}$ exposure in the destination neighborhoods of White, Black, Hispanic, Asian, poor, and nonpoor origin neighborhoods. I classify neighborhoods as poor and nonpoor based on whether the proportion of residents living under the federal poverty line was greater than 30%. I classified tracts as majority non-Hispanic White, non-Hispanic Black, non-Hispanic Asian, or Hispanic using a threshold of 50%. I tested different thresholds (40% for poor and 60% for race/ethnicity) and the general patterns do not significantly differ (*SI Appendix*, Figs. S1–S5). I run the following fixed-effects ordinary least-squares (OLS) regression model:

$$\begin{array}{l} WPM_{ik}=\beta_{0}+\beta_{1}W\mathrm{hit}e_{ik}+\beta_{2}B\mathrm{lac}k_{ik}+\beta_{3}A\mathrm{sia}n_{ik}+\beta_{4}Hisp_{ik}\\ +\beta_{5}Poor_{ik}+\beta_{6}Pop_{ik}+\alpha_{k}+\epsilon_{ik}, \end{array}$$

where *White_ik_*, *Black_ik_*, *Asian_ik_*, *Hisp_ik_*, and *Poor_ik_* are dummy variables indicating whether neighborhood *i* in city *k* is White, Black, Asian, Hispanic, or poor; *α_k_* is a city fixed effect, which controls for unobserved city characteristics; and *Pop_ik_* controls for differences in resident total population. Demographic data were obtained from the 2014 to 2018 American Community Survey. Descriptive statistics of all variables used in the study are provided in *SI Appendix*, Tables S1–S3.

**Fig. 1.**
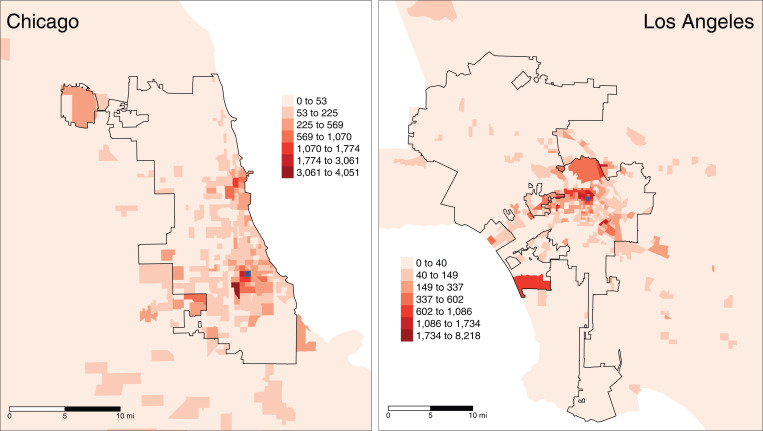
Neighborhoods shaded by the number of trips from representative neighborhoods (in blue) in Chicago and Los Angeles.

The outcome *WPM_ik_* is the average $PM_{2.5}$ levels in neighborhood *i*’s neighborhood mobility network weighted by the proportion of trips to each neighborhood in a city’s MSA. The matrix *W*, which has all city tracts represented in rows and all MSA tracts in columns, quantifies the spatial flows between a city’s neighborhoods and all neighborhoods within a city’s MSA. Cell *w_ij_* links tract *i* in the row to tract *j* in the column and represents the proportion of *i*’s total trips going to *j*. As a proportion, *w_ij_* indicates the strength of the association between *i* and *j* relative to all other neighborhoods in the MSA. Trips to the same neighborhood *i* (*w_ii_* = 0) were excluded. As an example of what the spatial distribution of flows might look like for a particular neighborhood, Fig. 1 shows the number of trips from single neighborhoods (in blue) in the South Side of Chicago and East Los Angeles. The city boundaries are in black, and MSA neighborhoods within and 4 miles beyond the city boundary are shown. One can see that trips are concentrated in neighborhoods sharing a border, but flows extend to nonadjacent areas and can reach quite far across the city and extend to neighborhoods outside of city boundaries within the MSA.

I also examined neighborhood racial and socioeconomic intersectional disparities in pollution exposure risk. Specifically, I ran the same fixed-effects model specified above but interacted the poverty indicator with each race/ethnicity indicator, yielding coefficients for indicators of nonpoor White, nonpoor Black, nonpoor Hispanic, nonpoor Asian, poor White, poor Black, poor Hispanic, and poor Asian neighborhoods. I excluded tracts that are missing values on any of the variables used in the study, have no neighbors sharing a border, have no trips leaving the neighborhood, and have no reported resident population. I also excluded cities where 90% or more of their neighborhoods were considered to be White, Black, Hispanic, Asian, poor, or nonpoor. I tested a threshold of 75% and results do not significantly differ (*SI Appendix*, Figs. S6–S12). These filters yielded a final analytic sample of 14,222 census tracts located in 88 cities.

## Results

**Fig. 2.**
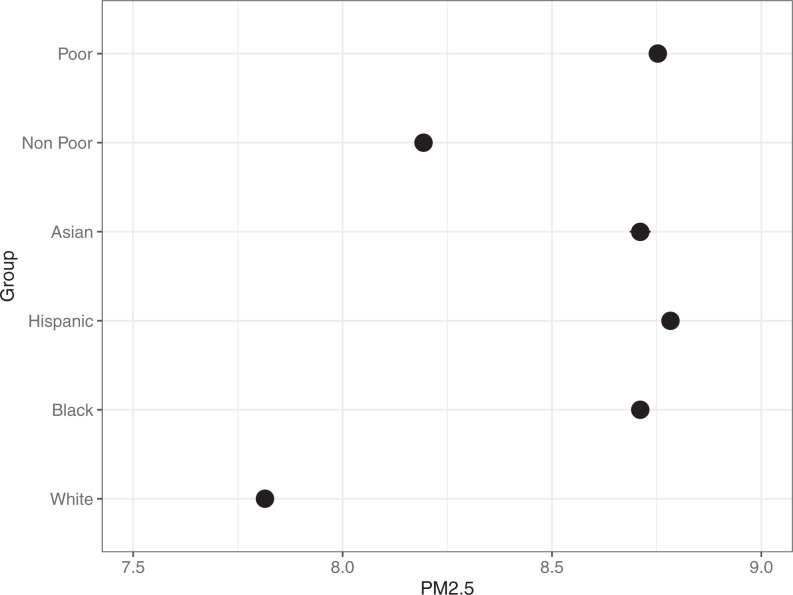
Regression-adjusted $PM_{2.5}$ (with 95% CIs) in the neighborhoods that residents travel to weighted by the number of trips.

Prior work has documented the disproportionately higher levels of exposure to environmental toxins in minority and poor neighborhoods (12). Do these disparities persist in the places that residents travel to outside of their residential neighborhood? To answer this question, Fig. 2 presents the regression-adjusted estimates and their 95% CIs of average $PM_{2.5}$ in a neighborhood’s mobility network weighted by the number of trips coming out of an origin neighborhood and traveling to a destination neighborhood (see *SI Appendix*, Tables S4–S8 for estimates, SEs, and 95% CIs). On average the neighborhoods that residents from non-White communities travel to have higher $PM_{2.5}$ levels than the neighborhoods connected to White communities. The $PM_{2.5}$ levels in Hispanic, Black, and Asian networks are 12.4% (8.78), 11.5% (8.71), and 11.5% (8.71) higher than the levels in White networks (7.81), respectively. Results also indicate disparities by neighborhood poverty. The neighborhoods that residents from poor neighborhoods travel to have average $PM_{2.5}$ levels that are 6.8% (8.75) higher than those of the neighborhoods that residents from nonpoor communities visit (8.19).

**Fig. 3.**
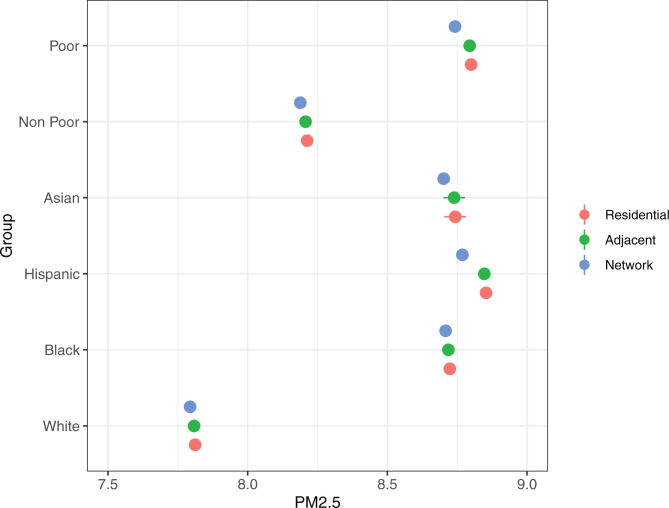
Regression-adjusted $PM_{2.5}$ (with 95% CIs) in residential neighborhoods (residential), adjacent neighborhoods (adjacent), and the nonadjacent neighborhoods that residents visit (network).

How do disparities at the neighborhood network level compare to those at the residential and extralocal levels? Fig. 3 presents the regression-adjusted estimates of average $PM_{2.5}$ in a neighborhood (residential), its bordering neighborhoods (adjacent), and the nonadjacent neighborhoods that residents visit (network). I examine only nonadjacent neighborhoods to exclude the possibility that the disparities presented in Fig. 2 are mainly driven by trips to adjacent areas. I find evidence of geographic clustering in $PM_{2.5}$, with residential neighborhoods surrounded by neighborhoods with similar $PM_{2.5}$ levels for all racial/ethnic and poverty groups. Results indicate that $PM_{2.5}$ levels are higher in minority residential neighborhoods compared to White residential neighborhoods. Average $PM_{2.5}$ exposures in Hispanic, Black, and Asian residential neighborhoods are 8.85, 8.72, and 8.74, respectively, compared to 7.81 in White residential neighborhoods. Neighborhoods adjacent to minority neighborhoods exhibit similar patterns (8.85, 8.72, and 8.74 in Hispanic, Black, and Asian adjacent neighborhoods, respectively, compared to 7.81 in White adjacent neighborhoods). Similar disparities exist between poor and nonpoor neighborhoods, with higher $PM_{2.5}$ levels in poor neighborhoods (8.80) and their adjacent neighborhoods (8.79) relative to their nonpoor counterparts (8.21 and 8.21, respectively).

Comparing average $PM_{2.5}$ across residential, adjacent, and network levels, I find that exposure is lowest in the mobility networks of Hispanic, White, poor, and nonpoor neighborhoods. Average exposures in Hispanic and White neighborhood mobility networks are 1.0 and 0.3% lower, respectively, than average exposure in both residential and adjacent neighborhoods. Exposures in poor and nonpoor neighborhood mobility networks are 0.3 and 0.6% lower, respectively. In contrast, average $PM_{2.5}$ is similar across all three levels for Black and Asian neighborhoods. That is, within-MSA mobility leads to lower pollution exposure for residents from Hispanic, White, nonpoor, and poor neighborhoods, but no change for residents from Black and Asian neighborhoods.

As a result of lower $PM_{2.5}$ exposure in White mobility networks but similar exposure in Black and Asian mobility networks, Black/White and Asian/White disparities are highest at the network level. In other words, not only are residents from Black and Asian neighborhoods exposed to higher levels of environmental toxins in their residential and extralocal environments compared to residents from White neighborhoods, but also these disparities increase in the neighborhoods they visit. In contrast, Hispanic/White network disparities are lower: Average $PM_{2.5}$ in Hispanic neighborhood networks (8.77) is 12.5% higher than in White neighborhood networks (7.79), whereas the difference is 13.3% (8.85 vs. 7.81) in both residential and adjacent neighborhoods. Similarly, average $PM_{2.5}$ in poor networks (8.74) is 6.7% higher than in nonpoor networks (8.19), but is 7.2% (8.80 vs. 8.21) and 7.1% (8.79 vs. 8.21) higher at the residential and adjacent levels, respectively.

**Fig. 4.**
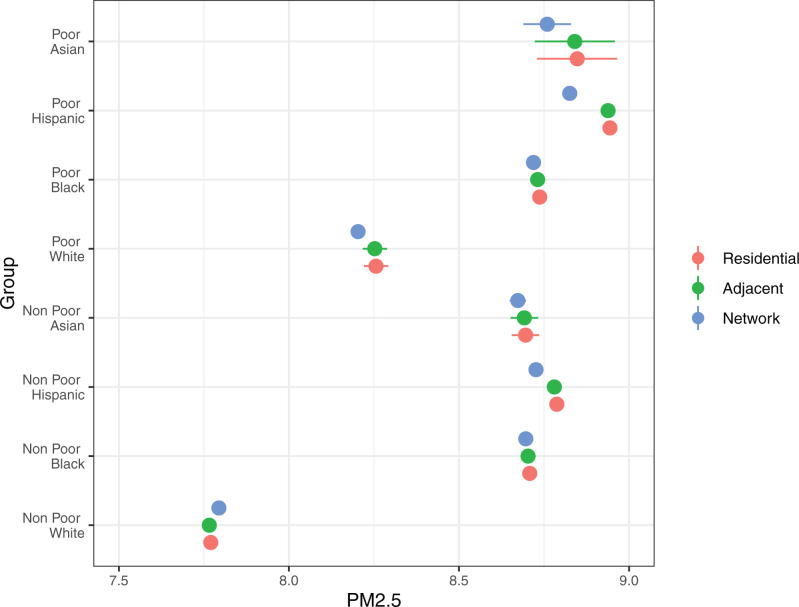
Regression-adjusted $PM_{2.5}$ (with 95% CIs) in residential neighborhoods (residential), adjacent neighborhoods (adjacent), and the nonadjacent neighborhoods that residents visit (network) by race/ethnicity and poverty status.

I next examine intersectional disparities in pollution exposure across neighborhood race/ethnicity and class. Results indicate that average $PM_{2.5}$ is lower in nonpoor neighborhoods relative to poor neighborhoods across residential, adjacent, and network levels for all racial/ethnic groups (Fig. 4). These results indicate a socioeconomic advantage in exposure risk; however, the advantage is much greater for White neighborhoods. Average $PM_{2.5}$ levels are 5.9, 5.9, and 5.2% lower in White nonpoor residential, adjacent, and network neighborhoods, respectively, than in poor neighborhoods. The advantages for minority nonpoor neighborhoods are considerably lower. For example, average $PM_{2.5}$ levels in Hispanic nonpoor residential, adjacent, and network neighborhoods are 1.8, 1.8, and 1.1% lower, respectively, than in their Hispanic poor counterparts. This greater White socioeconomic advantage results in much larger racial/ethnic disparities in exposure risk in nonpoor settings.

When comparing the exposure risk of poor and nonpoor neighborhoods across the three scales, racial/ethnic patterns resemble those shown in Fig. 3. Regardless of poverty status, average $PM_{2.5}$ is similar across Black and Asian residential, adjacent, and network levels. Hispanic mobility networks exhibit lower pollution exposure than Hispanic residential and adjacent neighborhoods in both poor and nonpoor settings; however, the decrease is much larger when the neighborhoods are poor. For example, the average $PM_{2.5}$ in Hispanic poor networks is 1.3% (8.83) lower than in Hispanic poor residential neighborhoods (8.94). The decreased exposure for Hispanic nonpoor mobility networks is smaller (0.7%; 8.73 vs. 8.79). In the case of White networks, I find that their overall lower exposure risk is driven by lower exposure in poor neighborhoods (0.6%; 8.20 vs. 8.26); there is a slight increase in average $PM_{2.5}$ exposure in network compared to residential and adjacent levels for White nonpoor neighborhoods (0.2%; 7.79 vs. 7.77).

**Fig. 5.**
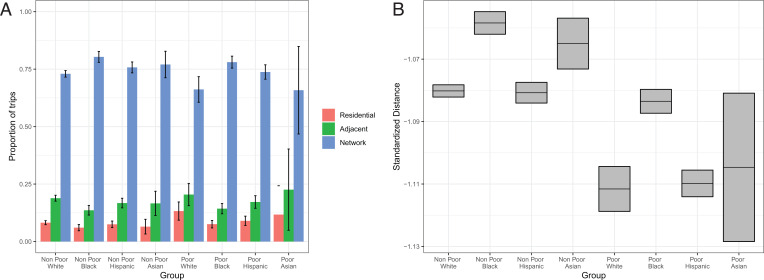
Within-MSA travel patterns by neighborhood racial/ethnic and poverty type (with 95% CIs). (*A*) Proportion of trips. (*B*) Average distance traveled.

The study’s findings indicate that racial/ethnic and socioeconomic disparities in pollution exposure extend beyond the residential and extralocal environments to the neighborhoods that residents visit. Fig. 5*A* shows that travel to these neighborhoods is substantial. Fig. 5*A* presents estimates from binomial regression models of the predicted proportions of visits staying within the residential neighborhood and going to adjacent and nonadjacent neighborhoods (OLS models yield similar results; *SI Appendix*, Fig. S11). Visits staying within the residential neighborhood include those to any block group within the residential census tract. Across all racial/ethnic and poverty neighborhood types, the majority of trips are to nonadjacent neighborhoods. Furthermore, residents from minority and poor neighborhoods travel outside of the residential and extralocal environments as much if not more than residents from White and nonpoor neighborhoods. Travel to nonadjacent neighborhoods is greatest for residents from Black neighborhoods, a finding that aligns with prior work using survey and social media data (28, 37). The proportions of trips that residents from poor Black neighborhoods that are within residential and go to adjacent and nonadjacent neighborhoods are 7.6, 14.3, and 78.0%, respectively. In comparison, these proportions are 13.3, 20.4, and 66.2%, respectively, for residents from poor White neighborhoods. Similar differences exist between nonpoor Black and White neighborhoods. Residents from Asian neighborhoods, whether poor or nonpoor, visit nonadjacent neighborhoods as often as residents from White neighborhoods, whereas residents from Hispanic neighborhoods travel more often within their mobility network.

Similar patterns hold when examining travel based on average distance (Fig. 5*B*), where distance is measured as the average Euclidean distance between the centroids of origin and destination neighborhoods weighted by the number of trips and standardized within MSAs to control for differences in area (full distribution of travel distances is provided in *SI Appendix*, Fig. S12). Residents from minority neighborhoods travel as far as or farther than residents from White neighborhoods in both poor and nonpoor settings. Residents from Black neighborhoods, whether poor or nonpoor, generally travel the farthest. Residents from Hispanic and White neighborhoods travel similar distances across poor and nonpoor settings, whereas residents from Asian poor neighborhoods travel similar distances to those of residents from White poor neighborhoods, but travel farther when their neighborhoods are not poor.

## Conclusion

Researchers studying neighborhood effects have provided convincing evidence that neighborhood conditions matter in an individual’s health and well-being. In particular, studies have established that minority and poor neighborhoods are exposed to higher levels of environmental hazards, which helps explain racial and socioeconomic disparities in individual outcomes (6, 12). Furthermore, this disadvantage extends to the areas that border minority and poor neighborhoods. My findings highlight another source of spatial inequality: the neighborhoods that city residents travel to within an MSA for work, errands, and leisure. Results indicate that residents from minority and poor neighborhoods visit neighborhoods that have greater air pollution levels than the neighborhoods that residents from White and nonpoor neighborhoods visit. The implications of this finding for health at the population level may be considerable given the ubiquity of ambient exposure to $PM_{2.5}$. For example, a study of a large cohort of adults in the United States found that an increase of 10 (μg/${\text{m}}^{3}$) $PM_{2.5}$ is associated with a 15% increase in cardiovascular disease mortality (38).

Hispanic neighborhoods carry the greatest pollution exposure burden. Whether poor or nonpoor, they have the highest levels of air pollution, they are next to neighborhoods that are similarly hazardous, and their residents travel to nonadjacent neighborhoods with the highest risk of exposure. However, the pollution levels in the neighborhoods that residents from Hispanic neighborhoods visit are lower than in the neighborhoods they live in. In contrast, exposure risk for residents from Black and Asian neighborhoods is similar across all levels. As a result of this higher exposure risk for residents of Black and Asian neighborhoods and lower exposure risk for residents of White neighborhoods, White/Black and White/Asian disparities are greatest at the network level. Furthermore, although nonpoor neighborhoods have lower pollution exposure than poor neighborhoods across all racial/ethnic groups, this decreased exposure is much greater for White neighborhoods. Overall, the results reveal that residents living in minority and poor neighborhoods face environmental inequalities at three scales: the neighborhoods they live in, adjacent neighborhoods, and the neighborhoods they visit. While prior work has provided extensive evidence of inequality in the first two settings, this study is one of the few to document it at the network level for a large sample of cities and to do so for risk exposure to $PM_{2.5}$.

Several caveats and areas of future research should be noted. First, census tracts in some cases may be large enough to have within-neighborhood differential exposure to air pollution. In these cases, analyses at a lower geographic level, such as the block group, are more appropriate. Second, the value of examining an outcome such as air pollution is that unlike traditional social metrics of neighborhood disadvantage such as poverty and the education levels of residents, exposure to pollution is direct (6). Nevertheless, disparities in associated health impacts could also reflect racial and socioeconomic variability in mobility, microenvironment, outdoor-to-indoor concentration relationships, dose–response, and access to health care, among other factors. Third, as the findings pertain to data at the neighborhood level, this study does not make claims about individuals’ travel patterns based on their particular race or class. That is, the study makes conclusions about minority and poor neighborhoods, but not minority and poor individuals. Fourth, the data reflect counts of unique devices visiting a location in a single day and capture only trips to points of interest. Fifth, as my data are measured from mobile phone usage, between-neighborhood comparisons could reflect differences in the likelihood that a resident owns and travels with a smart phone.

Although racial and income segregation and overall exposure to air pollution levels in the United States have decreased, significant disparities still exist between racial and socioeconomic groups (39, 40). This study demonstrates that racial and economic disparities in exposure to environmental hazards reach well beyond one’s home. That is, environmental inequalities are operating at a higher-order level than typically recognized: Unequal exposure is manifest not only where people live and the neighborhoods surrounding their residential settings but also where they travel throughout a city. In light of prior research showing that residents spend large proportions of time outside of their neighborhoods, this time is spent in distal areas of the city, and residents of poor and minority neighborhoods travel about as widely across their cities as those of other groups, the disparities uncovered in this study emphasize the importance of considering the network of neighborhoods connected via urban mobility when understanding neighborhood inequality (28, 37). The scholarly implication here is that by focusing exclusively on the residential neighborhood and its extralocal environs, prior work has largely underestimated the levels of spatial racial inequality in neighborhood disadvantage. The practical implication here is that policymakers should consider a neighborhood’s larger geographic and network community in developing interventions to counter the multilayer nature of urban spatial inequality. For example, practitioners from network-connected neighborhoods can collaborate to share and deploy resources across their respective communities such as increasing public transit between neighborhoods, which will reduce overall air pollution levels within the existing network cluster, and to less disadvantaged, low pollution areas outside of the network, particularly those at longer distances. Adopting a network perspective can also increase efficiency in resource allocation by focusing interventions in the most polluted and visited neighborhoods within a mobility network.

## Supplementary Material

Supplementary File

## Data Availability

Anonymized comma-separated values data have been deposited in https://github.com/nbrazil/envinequality.
