# Supplementary material for: Environmental inequality in the neighborhood networks of urban mobility in US cities
Source: Proc Natl Acad Sci U S A. 2022 Apr 21;119(17):e2117776119. doi: 10.1073/pnas.2117776119 (PMC9169920; doi:10.1073/pnas.2117776119)
Supplement: Supplementary File [file pnas.2117776119.sapp.pdf]

PNAS
www.pnas.org

Supplementary Information for

Environmental inequality in the neighborhood networks of urban mobility in US cities

Noli Brazil

Noli Brazil
Email: nbrazil@ucdavis.edu

This PDF file includes:

Figures S1 to S12
Tables S1 to S8

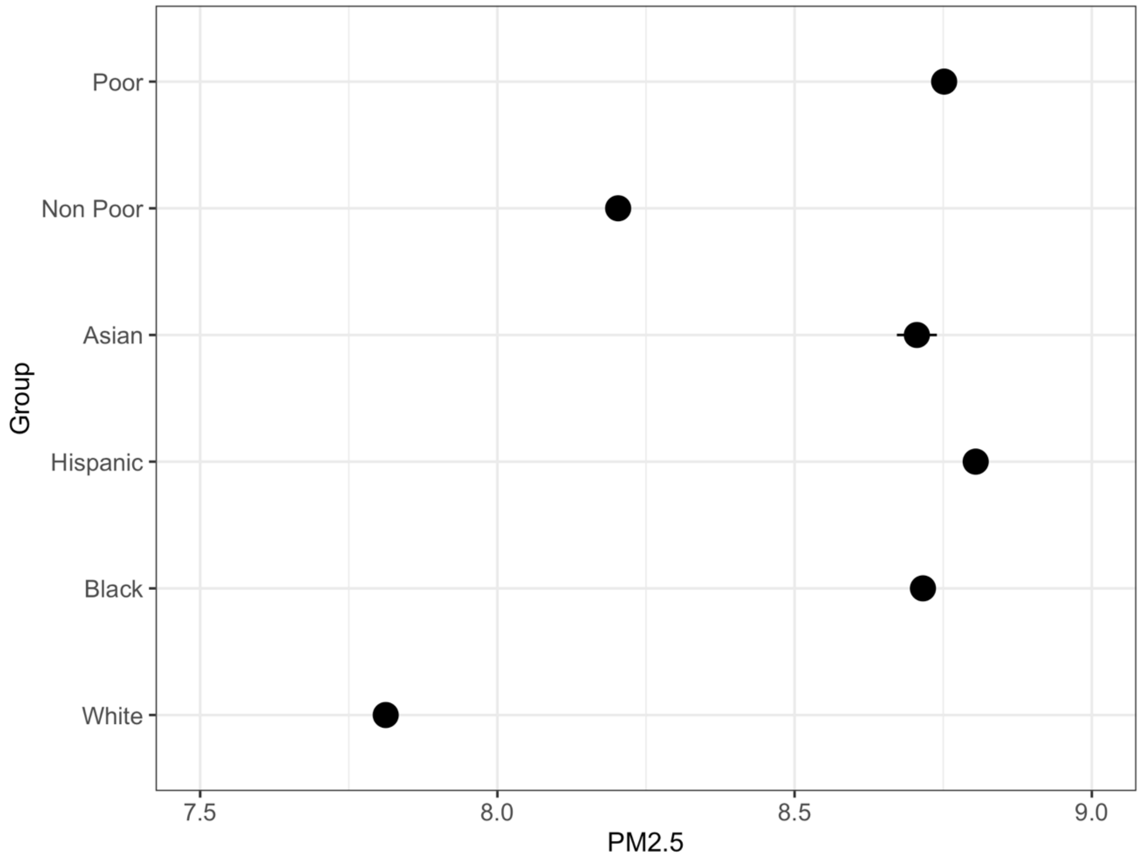

Fig. S1. Regression adjusted $PM_{2.5}$ (with 95% confidence intervals) in the neighborhoods that residents travel to weighted by the number of trips, 60% race/ethnicity and 40% poverty thresholds

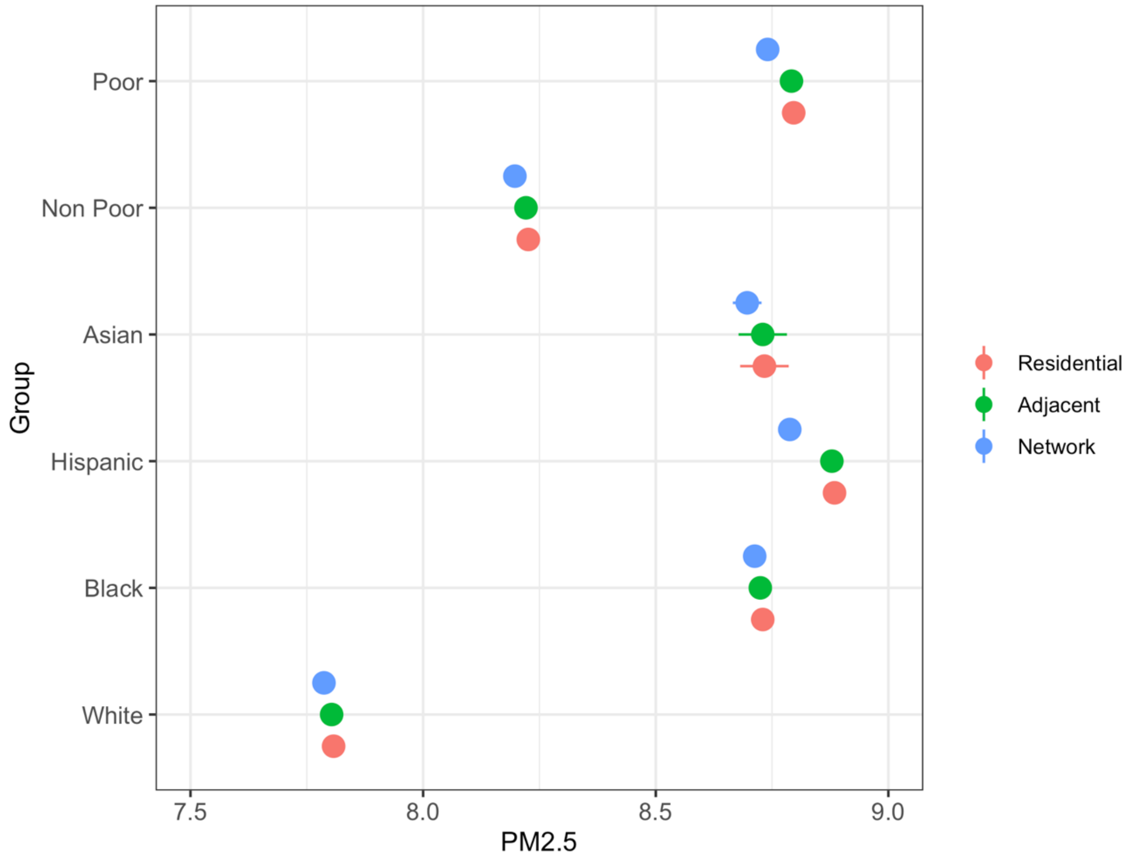

Fig. S2. Regression adjusted $PM_{2.5}$ (with 95% confidence intervals) in residential neighborhoods (Residential), adjacent neighborhoods (Adjacent) and the non-adjacent neighborhoods that residents travel to (Network) weighted by the number of trips, 60% race/ethnicity and 40% poverty thresholds

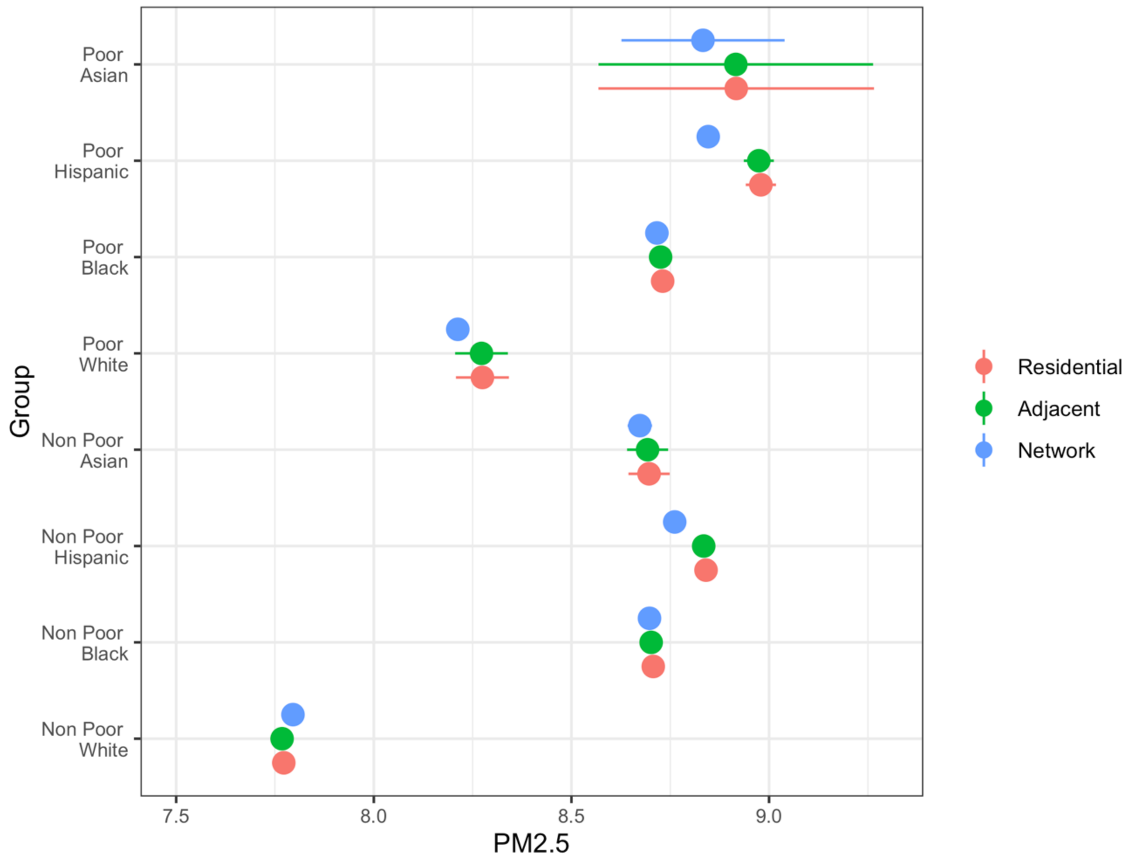

Fig. S3. Regression adjusted $PM_{2.5}$ (with 95% confidence intervals) in residential neighborhoods (Residential), adjacent neighborhoods (Adjacent) and the non-adjacent neighborhoods that residents travel to (Network) weighted by the number of trips by race/ethnicity and poverty status, 60% race/ethnicity and 40% poverty thresholds

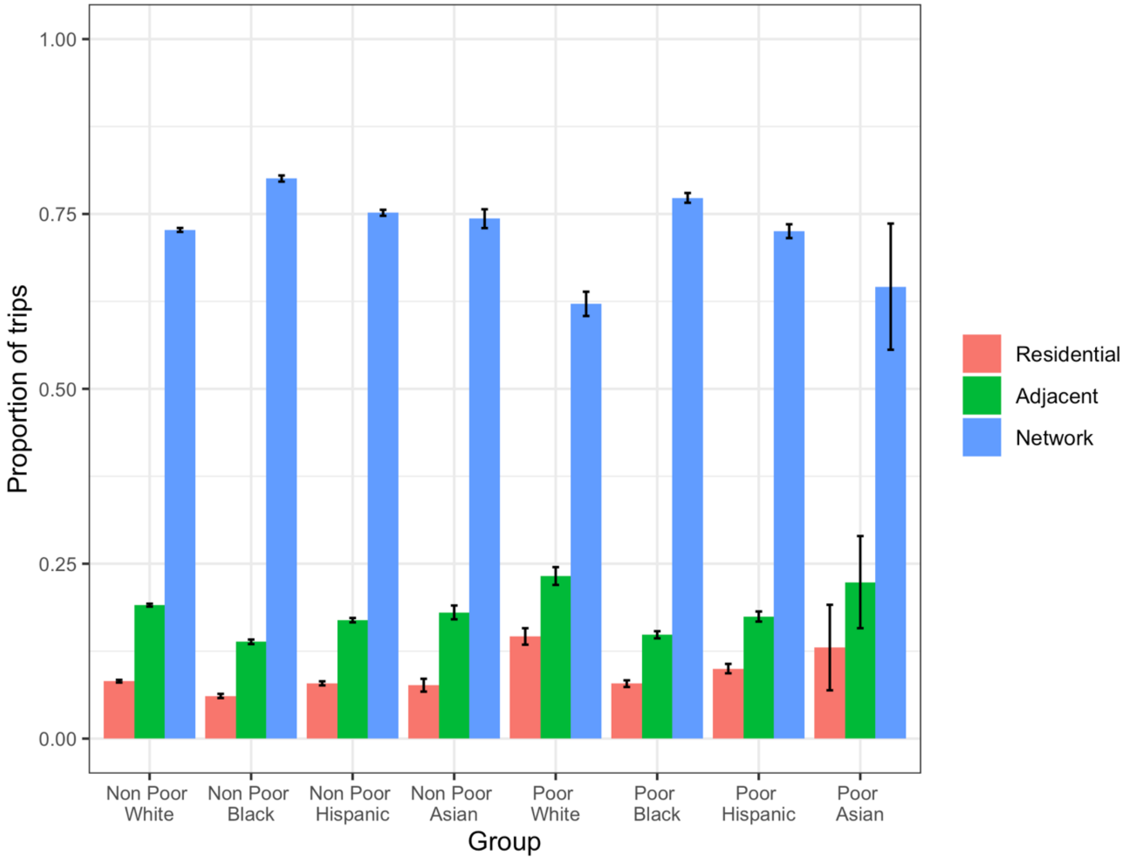

Fig. S4. Within-MSA travel patterns by neighborhood racial/ethnic and poverty type, Proportion of trips (with 95% confidence intervals), 60% race/ethnicity and 40% poverty thresholds

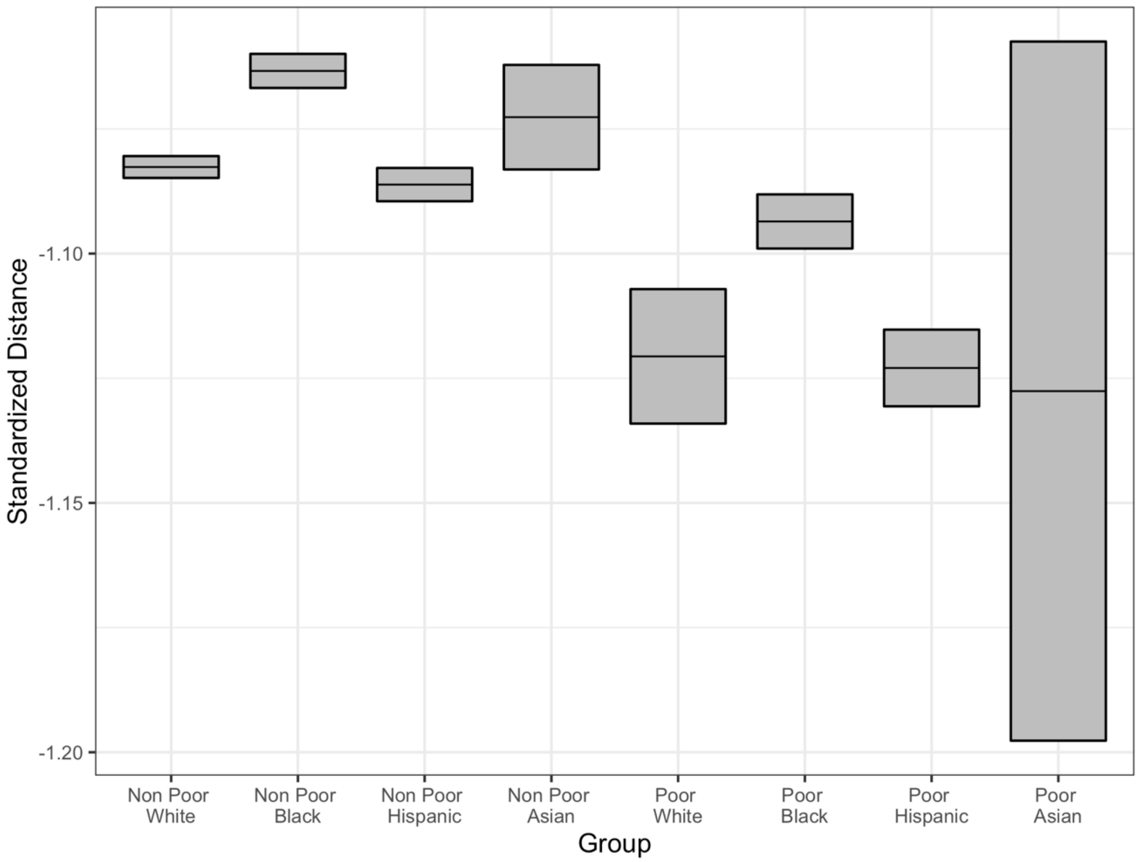

Fig. S5. Within-MSA travel patterns by neighborhood racial/ethnic and poverty type, Average distance travelled (with 95% confidence intervals), 60% race/ethnicity and 40% poverty thresholds

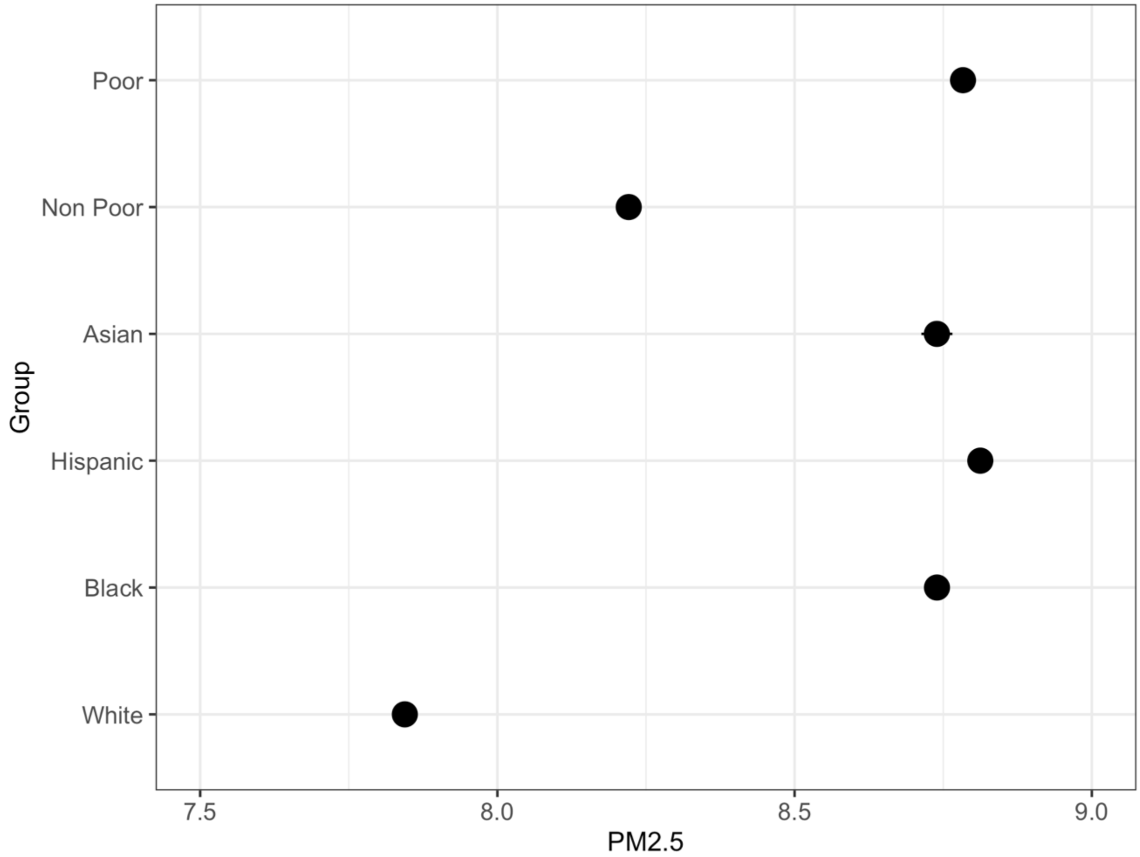

Fig. S6. Regression adjusted $PM_{2.5}$ (with 95% confidence intervals) in the neighborhoods that residents travel to weighted by the number of trips, excludes cities with 75% or more of their neighborhoods considered to be White, Black, Hispanic, Asian, Poor or Nonpoor

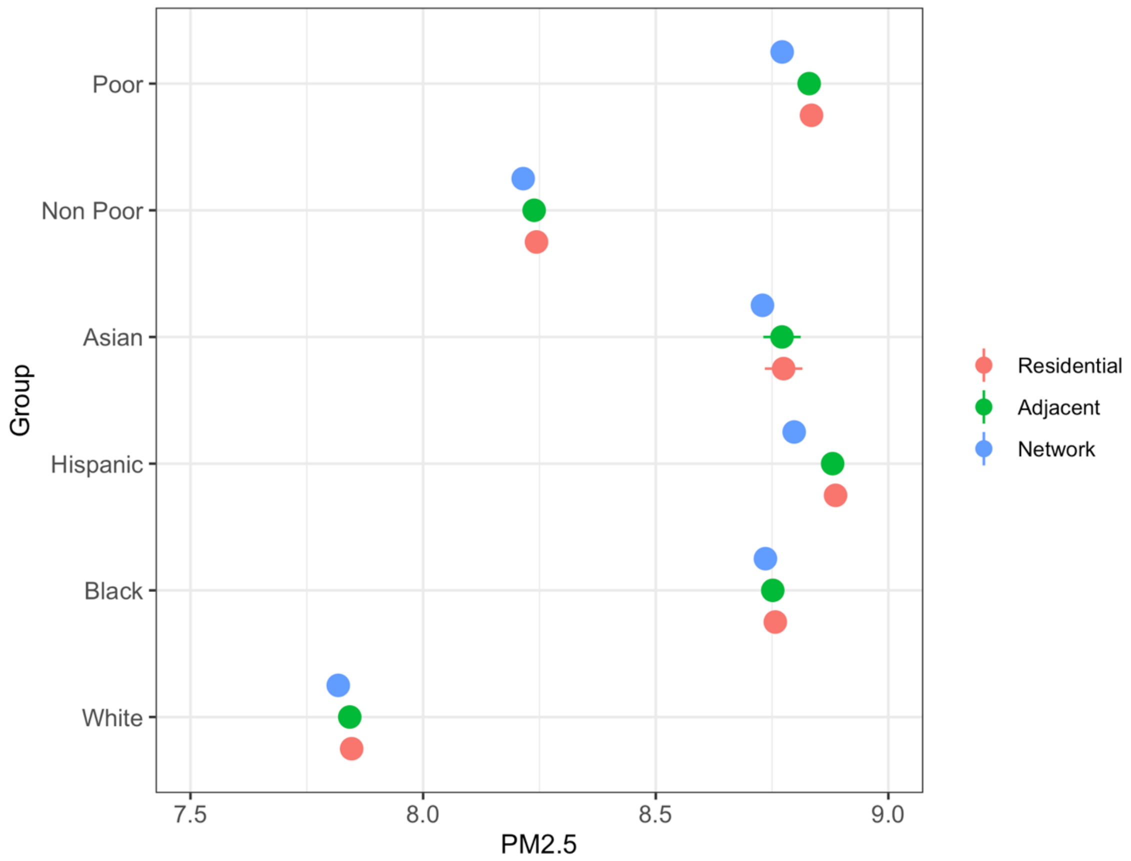

Fig. S7. Regression adjusted $PM_{2.5}$ (with 95% confidence intervals) in residential neighborhoods (Residential), adjacent neighborhoods (Adjacent) and the non-adjacent neighborhoods that residents travel to (Network) weighted by the number of trips, excludes cities with 75% or more of their neighborhoods considered to be White, Black, Hispanic, Asian, Poor or Nonpoor

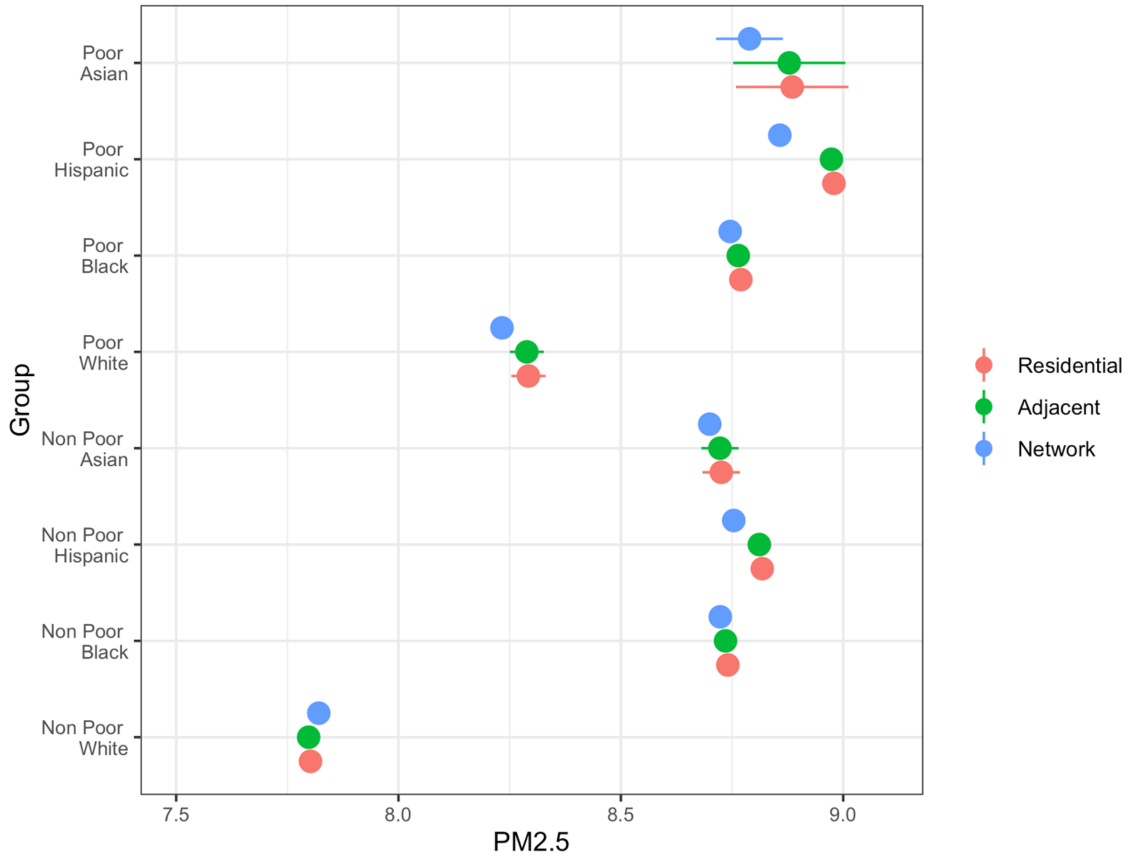

Fig. S8. Regression adjusted $PM_{2.5}$ (with 95% confidence intervals) in residential neighborhoods (Residential), adjacent neighborhoods (Adjacent) and the non-adjacent neighborhoods that residents travel to (Network) weighted by the number of trips by race/ethnicity and poverty status, excludes cities with 75% or more of their neighborhoods considered to be White, Black, Hispanic, Asian, Poor or Nonpoor

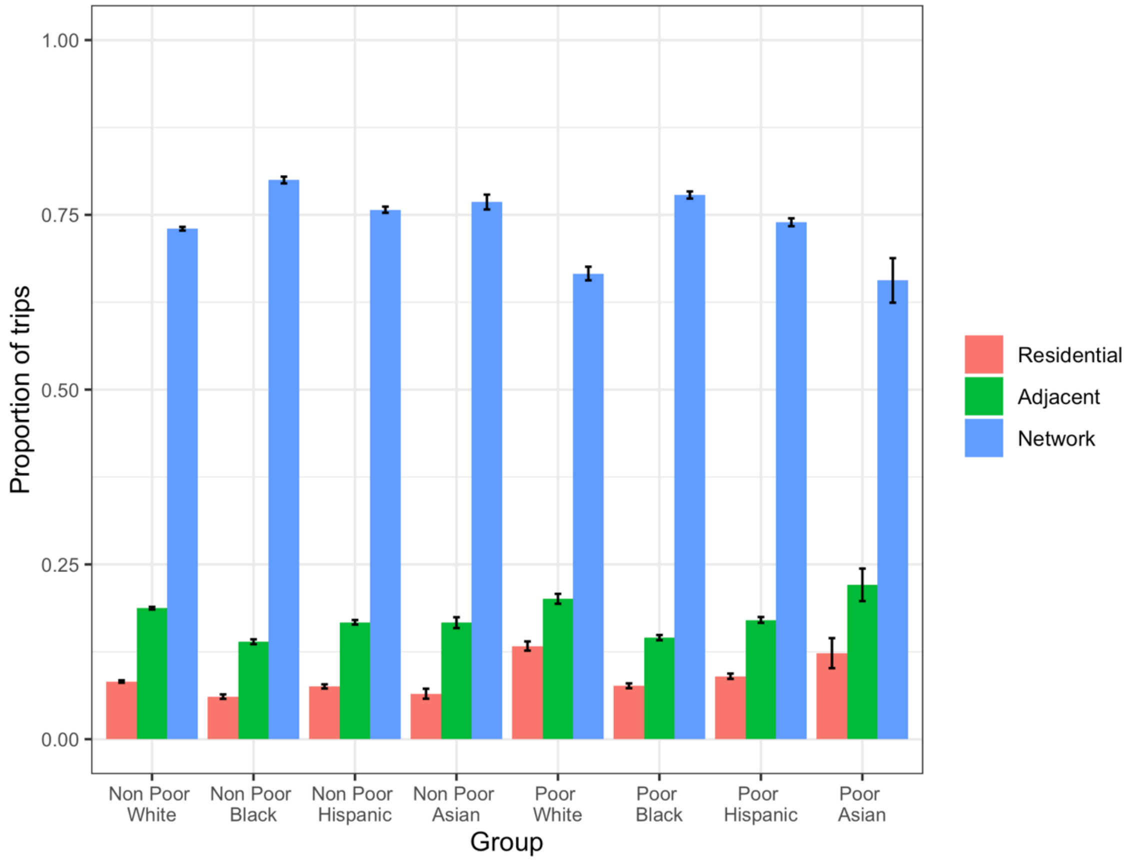

Fig. S9. Within-MSA travel patterns by neighborhood racial/ethnic and poverty type, Proportion of trips (with 95% confidence intervals), excludes cities with 75% or more of their neighborhoods considered to be White, Black, Hispanic, Asian, Poor or Nonpoor

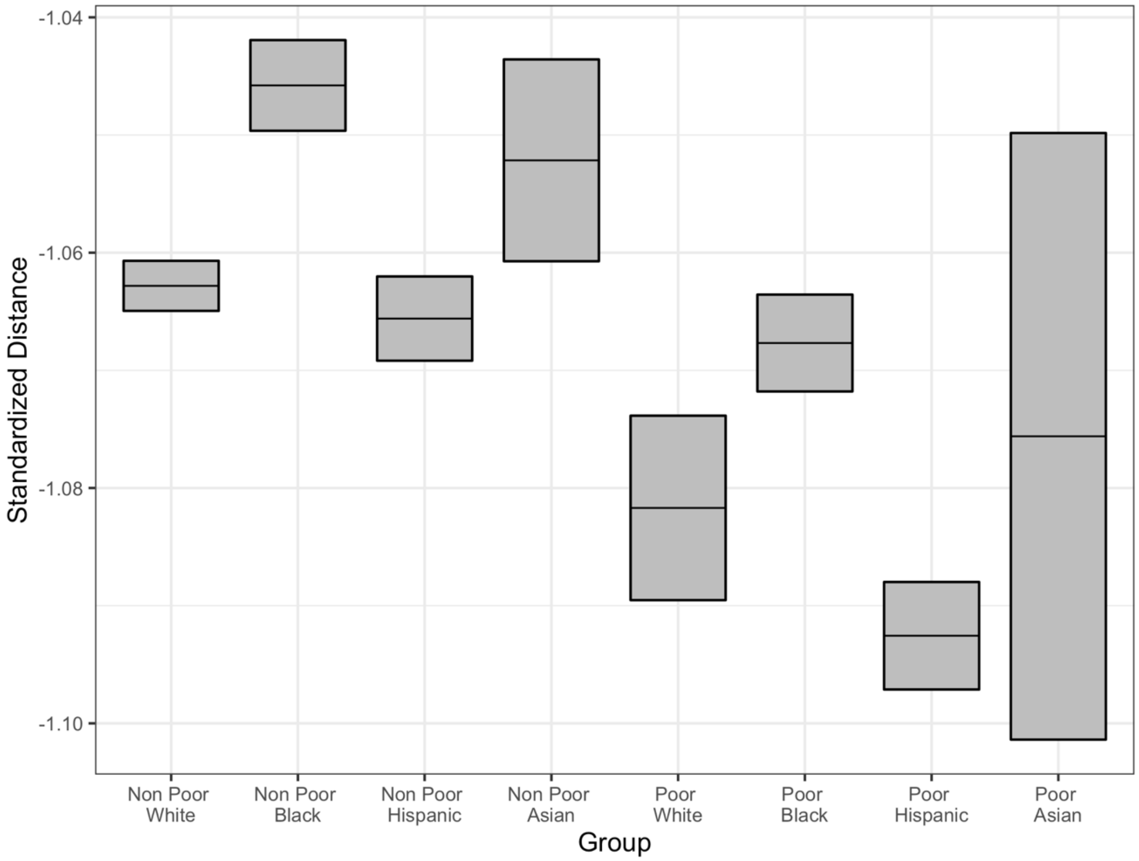

Fig. S10. Within-MSA travel patterns by neighborhood racial/ethnic and poverty type, Average distance travelled (with 95% confidence intervals), excludes cities with 75% or more of their neighborhoods considered to be White, Black, Hispanic, Asian, Poor or Nonpoor

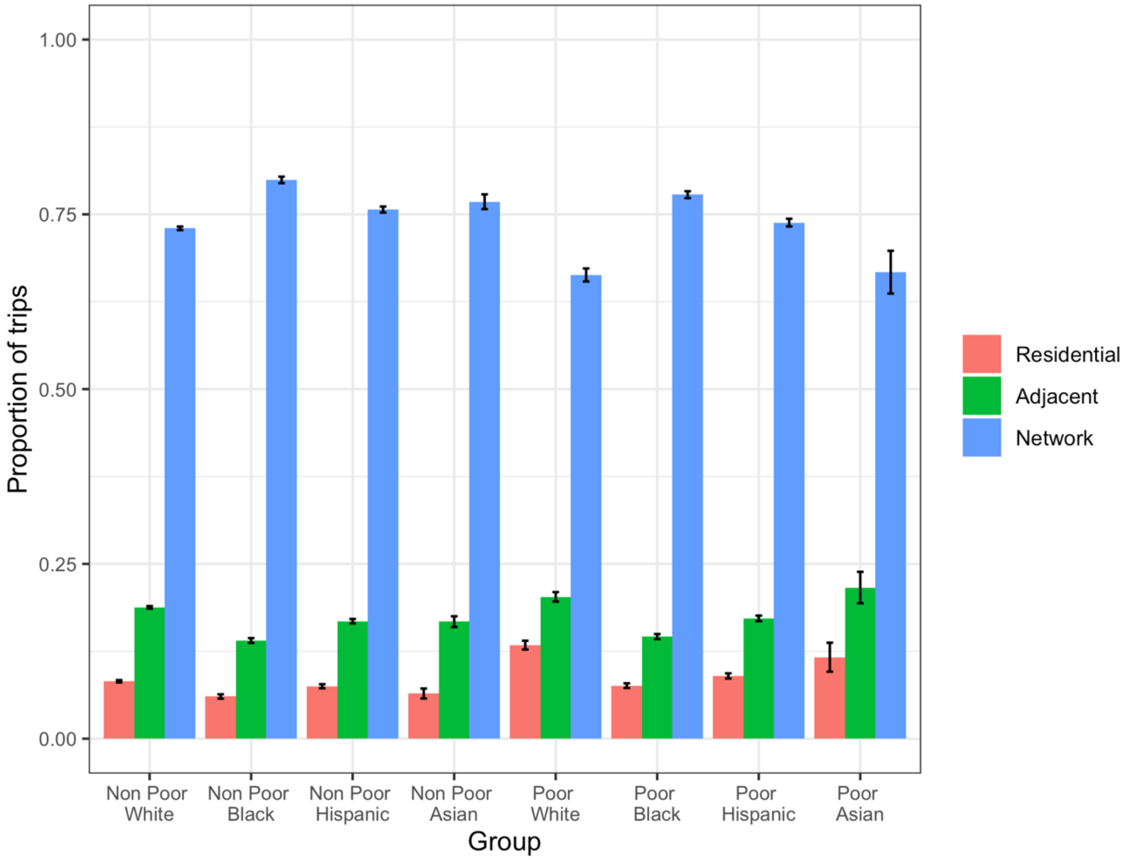

Fig. S11. Within-MSA travel patterns by neighborhood racial/ethnic and poverty type, Ordinary Least Squares regression (with 95% confidence intervals).

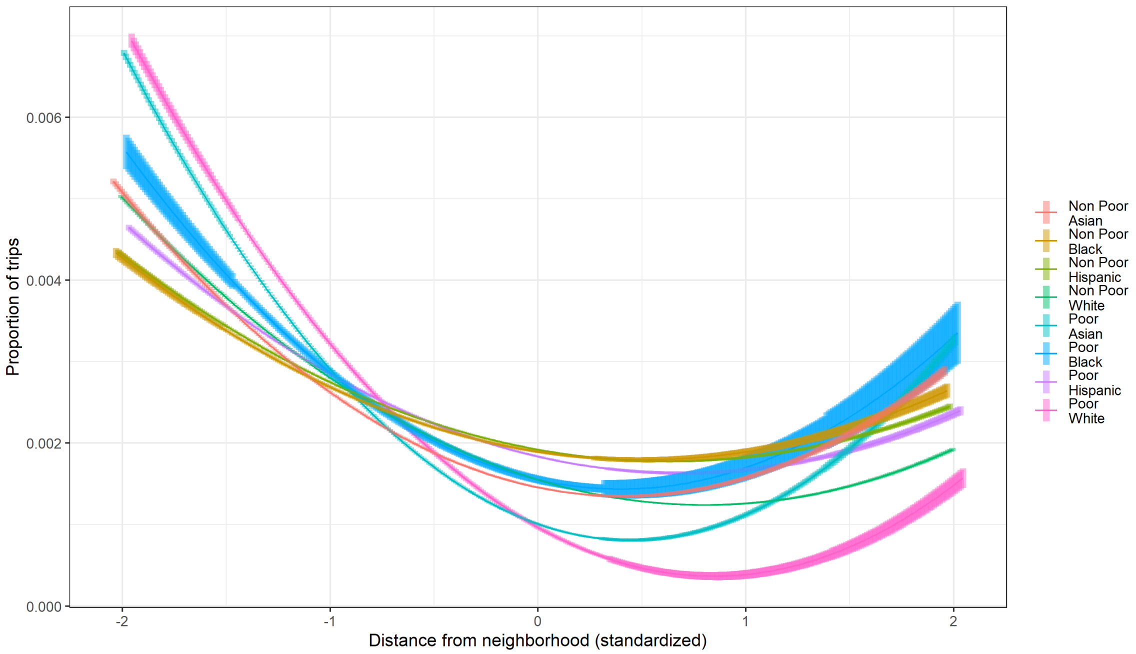

Fig. S12. Within-MSA travel patterns by neighborhood racial/ethnic and poverty type, distribution of distance travelled, Ordinary Least Squares regression with quadratic distance (with 95% confidence intervals).

Table S1. Descriptive statistics (N = 14,222)

Variable	Mean	SD	Min	Max
Race/ethnic neighborhood type				
Asian	0.02	0.14	0.00	1.00
Black	0.19	0.40	0.00	1.00
Hispanic	0.19	0.39	0.00	1.00
White	0.38	0.48	0.00	1.00
Poverty neighborhood type				
Poor	0.23	0.42	0.00	1.00
Race/ethnic by poverty neighborhood type				
Nonpoor				
Asian	0.02	0.13	0.00	1.00
Black	0.10	0.30	0.00	1.00
Hispanic	0.13	0.33	0.00	1.00
White	0.36	0.48	0.00	1.00
Poor				
Asian	0.00	0.04	0.00	1.00
Black	0.10	0.29	0.00	1.00
Hispanic	0.07	0.25	0.00	1.00
White	0.02	0.14	0.00	1.00
Total population	4,100	2,098	3	32,326
Total trips	59,749	42,100	16	1,003,248
Distance travelled (standardized)	-1.12	0.18	-1.60	-0.08
Proportion of trips				
Residential	0.08	0.06	0.00	0.56
Adjacent	0.16	0.07	0.00	0.61
Neighborhood network (non-adjacent)	0.77	0.09	0.24	1.00
PM25				
Residential	8.89	1.36	4.21	13.54
Adjacent	8.88	1.35	4.20	13.51
Neighborhood network (all)	8.87	1.32	5.34	13.03
Neighborhood network (non-adjacent)	8.87	1.31	5.37	12.97

Table S2. List of cities and their Metropolitan Statistical Areas (N = 88)

City	Metropolitan Statistical Areas
Albuquerque, New Mexico	Albuquerque, NM
Anaheim, California	Los Angeles-Long Beach-Anaheim, CA
Arlington, Texas	Dallas-Fort Worth-Arlington, TX
Atlanta, Georgia	Atlanta-Sandy Springs-Roswell, GA
Aurora, Colorado	Denver-Aurora-Lakewood, CO
Austin, Texas	Austin-Round Rock, TX
Bakersfield, California	Bakersfield, CA
Baltimore, Maryland	Baltimore-Columbia-Towson, MD
Baton Rouge, Louisiana	Baton Rouge, LA
Boston, Massachusetts	Boston-Cambridge-Newton, MA-NH
Buffalo, New York	Buffalo-Cheektowaga-Niagara Falls, NY
Chandler, Arizona	Phoenix-Mesa-Scottsdale, AZ
Charlotte, North Carolina	Charlotte-Concord-Gastonia, NC-SC
Chesapeake, Virginia	Virginia Beach-Norfolk-Newport News, VA-NC
Chicago, Illinois	Chicago-Naperville-Elgin, IL-IN-WI
Chula Vista, California	San Diego-Carlsbad, CA
Cincinnati, Ohio	Cincinnati, OH-KY-IN
Cleveland, Ohio	Cleveland-Elyria, OH
Colorado Springs, Colorado	Colorado Springs, CO
Columbus, Ohio	Columbus, OH
Corpus Christi, Texas	Corpus Christi, TX
Dallas, Texas	Dallas-Fort Worth-Arlington, TX
Denver, Colorado	Denver-Aurora-Lakewood, CO
Des Moines, Iowa	Des Moines-West Des Moines, IA

Detroit, Michigan	Detroit-Warren-Dearborn, MI
Durham, North Carolina	Durham-Chapel Hill, NC
Fort Wayne, Indiana	Fort Wayne, IN
Fort Worth, Texas	Dallas-Fort Worth-Arlington, TX
Fremont, California	San Francisco-Oakland-Hayward, CA
Fresno, California	Fresno, CA
Garland, Texas	Dallas-Fort Worth-Arlington, TX
Glendale, Arizona	Phoenix-Mesa-Scottsdale, AZ
Greensboro, North Carolina	Greensboro-High Point, NC
Houston, Texas	Houston-The Woodlands-Sugar Land, TX
Indianapolis (balance), Indiana	Indianapolis-Carmel-Anderson, IN
Irvine, California	Los Angeles-Long Beach-Anaheim, CA
Irving, Texas	Dallas-Fort Worth-Arlington, TX
Jacksonville, Florida	Jacksonville, FL
Jersey, New Jersey	New York-Newark-Jersey City, NY-NJ-PA
Kansas, Missouri	Kansas City, MO-KS
Las Vegas, Nevada	Las Vegas-Henderson-Paradise, NV
Long Beach, California	Los Angeles-Long Beach-Anaheim, CA
Los Angeles, California	Los Angeles-Long Beach-Anaheim, CA
Louisville, Kentucky	Louisville/Jefferson County, KY-IN
Lubbock, Texas	Lubbock, TX
Memphis, Tennessee	Memphis, TN-MS-AR
Mesa, Arizona	Phoenix-Mesa-Scottsdale, AZ
Miami, Florida	Miami-Fort Lauderdale-West Palm Beach, FL
Milwaukee, Wisconsin	Milwaukee-Waukesha-West Allis, WI
Minneapolis, Minnesota	Minneapolis-St. Paul-Bloomington, MN-WI
Nashville, Tennessee	Nashville-Davidson--Murfreesboro--Franklin, TN

New Orleans, Louisiana	New Orleans-Metairie, LA
New York, New York	New York-Newark-Jersey City, NY-NJ-PA
Newark, New Jersey	New York-Newark-Jersey City, NY-NJ-PA
Norfolk, Virginia	Virginia Beach-Norfolk-Newport News, VA-NC
North Las Vegas, Nevada	Las Vegas-Henderson-Paradise, NV
Oakland, California	San Francisco-Oakland-Hayward, CA
Oklahoma, Oklahoma	Oklahoma City, OK
Omaha, Nebraska	Omaha-Council Bluffs, NE-IA
Orlando, Florida	Orlando-Kissimmee-Sanford, FL
Philadelphia, Pennsylvania	Philadelphia-Camden-Wilmington, PA-NJ-DE-MD
Phoenix, Arizona	Phoenix-Mesa-Scottsdale, AZ
Pittsburgh, Pennsylvania	Pittsburgh, PA
Plano, Texas	Dallas-Fort Worth-Arlington, TX
Raleigh, North Carolina	Raleigh, NC
Reno, Nevada	Reno, NV
Richmond, Virginia	Richmond, VA
Riverside, California	Riverside-San Bernardino-Ontario, CA
Sacramento, California	Sacramento--Roseville--Arden-Arcade, CA
San Antonio, Texas	San Antonio-New Braunfels, TX
San Bernardino, California	Riverside-San Bernardino-Ontario, CA
San Diego, California	San Diego-Carlsbad, CA
San Francisco, California	San Francisco-Oakland-Hayward, CA
San Jose, California	San Jose-Sunnyvale-Santa Clara, CA
Santa Ana, California	Los Angeles-Long Beach-Anaheim, CA
Seattle, Washington	Seattle-Tacoma-Bellevue, WA
St. Louis, Missouri	St. Louis, MO-IL
St. Paul, Minnesota	Minneapolis-St. Paul-Bloomington, MN-WI

St. Petersburg, Florida	Tampa-St. Petersburg-Clearwater, FL
Stockton, California	Stockton-Lodi, CA
Tampa, Florida	Tampa-St. Petersburg-Clearwater, FL
Toledo, Ohio	Toledo, OH
Tucson, Arizona	Tucson, AZ
Tulsa, Oklahoma	Tulsa, OK
Virginia Beach, Virginia	Virginia Beach-Norfolk-Newport News, VA-NC
Washington, District of Columbia	Washington-Arlington-Alexandria, DC-VA-MD-WV
Wichita, Kansas	Wichita, KS
Winston-Salem, North Carolina	Winston-Salem, NC

Table S3. Descriptive statistics by city

	White			Black			Hispanic			Asian			Poor		
**City**	**25th**	**75th**	**Mean**	**25th**	**75th**	**Mean**	**25th**	**75th**	**Mean**	**25th**	**75th**	**Mean**	**25th**	**75th**	**Mean**
Albuquerque, New Mexico	0.00	1.00	0.35	0.00	0.00	0.00	0.00	1.00	0.33	0.00	0.00	0.00	0.00	0.00	0.14
Anaheim, California	0.00	0.00	0.16	0.00	0.00	0.00	0.00	1.00	0.63	0.00	0.00	0.00	0.00	0.00	0.04
Arlington, Texas	0.00	1.00	0.44	0.00	0.00	0.00	0.00	0.00	0.19	0.00	0.00	0.00	0.00	0.00	0.14
Atlanta, Georgia	0.00	1.00	0.37	0.00	1.00	0.54	0.00	0.00	0.00	0.00	0.00	0.00	0.00	1.00	0.38
Aurora, Colorado	0.00	1.00	0.42	0.00	0.00	0.00	0.00	0.00	0.14	0.00	0.00	0.00	0.00	0.00	0.03
Austin, Texas	0.00	1.00	0.58	0.00	0.00	0.00	0.00	0.00	0.22	0.00	0.00	0.00	0.00	0.00	0.11
Bakersfield, California	0.00	1.00	0.27	0.00	0.00	0.00	0.00	1.00	0.49	0.00	0.00	0.00	0.00	0.00	0.24
Baltimore, Maryland	0.00	0.00	0.22	0.00	1.00	0.65	0.00	0.00	0.00	0.00	0.00	0.00	0.00	1.00	0.31
Baton Rouge, Louisiana	0.00	1.00	0.38	0.00	1.00	0.58	0.00	0.00	0.00	0.00	0.00	0.00	0.00	1.00	0.47
Boston, Massachusetts	0.00	1.00	0.50	0.00	0.00	0.18	0.00	0.00	0.05	0.00	0.00	0.01	0.00	0.00	0.22
Buffalo, New York	0.00	1.00	0.44	0.00	1.00	0.33	0.00	0.00	0.01	0.00	0.00	0.00	0.00	1.00	0.53
Chandler, Arizona	1.00	1.00	0.89	0.00	0.00	0.00	0.00	0.00	0.07	0.00	0.00	0.00	0.00	0.00	0.05
Charlotte, North Carolina	0.00	1.00	0.37	0.00	1.00	0.27	0.00	0.00	0.01	0.00	0.00	0.00	0.00	0.00	0.15
Chesapeake, Virginia	0.00	1.00	0.73	0.00	0.00	0.20	0.00	0.00	0.00	0.00	0.00	0.00	0.00	0.00	0.02
Chicago, Illinois	0.00	1.00	0.30	0.00	1.00	0.35	0.00	0.00	0.21	0.00	0.00	0.01	0.00	0.00	0.25
Chula Vista, California	0.00	0.00	0.00	0.00	0.00	0.00	1.00	1.00	0.78	0.00	0.00	0.00	0.00	0.00	0.07
Cincinnati, Ohio	0.00	1.00	0.52	0.00	1.00	0.36	0.00	0.00	0.00	0.00	0.00	0.00	0.00	1.00	0.41
Cleveland, Ohio	0.00	0.00	0.22	0.00	1.00	0.54	0.00	0.00	0.02	0.00	0.00	0.00	0.00	1.00	0.68
Colorado Springs, Colorado	1.00	1.00	0.85	0.00	0.00	0.00	0.00	0.00	0.01	0.00	0.00	0.00	0.00	0.00	0.09
Columbus, Ohio	0.00	1.00	0.59	0.00	1.00	0.29	0.00	0.00	0.00	0.00	0.00	0.00	0.00	1.00	0.31
Corpus Christi, Texas	0.00	0.00	0.10	0.00	0.00	0.00	1.00	1.00	0.79	0.00	0.00	0.00	0.00	0.00	0.08
Dallas, Texas	0.00	1.00	0.30	0.00	0.00	0.15	0.00	1.00	0.32	0.00	0.00	0.00	0.00	0.00	0.21
Denver, Colorado	0.00	1.00	0.64	0.00	0.00	0.00	0.00	0.00	0.22	0.00	0.00	0.00	0.00	0.00	0.04

Des Moines, Iowa	1.00	1.00	0.84	0.00	0.00	0.00	0.00	0.00	0.00	0.00	0.00	0.00	0.00	0.00	0.11
Detroit, Michigan	0.00	0.00	0.02	1.00	1.00	0.87	0.00	0.00	0.05	0.00	0.00	0.00	0.00	1.00	0.73
Durham, North Carolina	0.00	1.00	0.36	0.00	0.50	0.26	0.00	0.00	0.00	0.00	0.00	0.00	0.00	0.00	0.21
Fort Wayne, Indiana	1.00	1.00	0.78	0.00	0.00	0.05	0.00	0.00	0.00	0.00	0.00	0.00	0.00	1.00	0.27
Fort Worth, Texas	0.00	1.00	0.31	0.00	0.00	0.10	0.00	1.00	0.28	0.00	0.00	0.00	0.00	0.00	0.18
Fremont, California	0.00	0.00	0.00	0.00	0.00	0.00	0.00	0.00	0.00	0.00	1.00	0.64	0.00	0.00	0.00
Fresno, California	0.00	0.00	0.22	0.00	0.00	0.00	0.00	1.00	0.53	0.00	0.00	0.00	0.00	1.00	0.44
Garland, Texas	0.00	0.00	0.07	0.00	0.00	0.00	0.00	1.00	0.38	0.00	0.00	0.00	0.00	0.00	0.05
Glendale, Arizona	0.00	1.00	0.54	0.00	0.00	0.00	0.00	0.75	0.26	0.00	0.00	0.00	0.00	0.00	0.17
Greensboro, North Carolina	0.00	1.00	0.46	0.00	1.00	0.28	0.00	0.00	0.00	0.00	0.00	0.00	0.00	0.00	0.18
Houston, Texas	0.00	0.00	0.23	0.00	0.00	0.15	0.00	1.00	0.38	0.00	0.00	0.00	0.00	0.00	0.24
Indianapolis (balance), Indiana	0.00	1.00	0.57	0.00	0.75	0.25	0.00	0.00	0.00	0.00	0.00	0.00	0.00	1.00	0.26
Irvine, California	0.00	1.00	0.36	0.00	0.00	0.00	0.00	0.00	0.00	0.00	0.00	0.09	0.00	0.00	0.12
Irving, Texas	0.00	0.00	0.02	0.00	0.00	0.00	0.00	1.00	0.53	0.00	0.00	0.08	0.00	0.00	0.06
Jacksonville, Florida	0.00	1.00	0.56	0.00	0.00	0.22	0.00	0.00	0.00	0.00	0.00	0.00	0.00	0.00	0.17
Jersey, New Jersey	0.00	0.00	0.08	0.00	0.00	0.12	0.00	0.00	0.06	0.00	0.00	0.09	0.00	0.00	0.11
Kansas, Missouri	0.00	1.00	0.52	0.00	1.00	0.32	0.00	0.00	0.04	0.00	0.00	0.00	0.00	0.00	0.21
Las Vegas, Nevada	0.00	1.00	0.48	0.00	0.00	0.01	0.00	0.00	0.20	0.00	0.00	0.00	0.00	0.00	0.16
Long Beach, California	0.00	0.25	0.25	0.00	0.00	0.01	0.00	1.00	0.40	0.00	0.00	0.00	0.00	0.00	0.18
Los Angeles, California	0.00	1.00	0.26	0.00	0.00	0.03	0.00	1.00	0.48	0.00	0.00	0.02	0.00	0.00	0.18
Louisville, Kentucky	1.00	1.00	0.89	0.00	0.00	0.04	0.00	0.00	0.01	0.00	0.00	0.00	0.00	0.00	0.03
Lubbock, Texas	0.00	1.00	0.62	0.00	0.00	0.04	0.00	0.00	0.16	0.00	0.00	0.00	0.00	1.00	0.33
Memphis, Tennessee	0.00	0.00	0.22	0.00	1.00	0.69	0.00	0.00	0.01	0.00	0.00	0.00	0.00	1.00	0.49
Mesa, Arizona	0.00	1.00	0.72	0.00	0.00	0.00	0.00	0.00	0.13	0.00	0.00	0.00	0.00	0.00	0.10
Miami, Florida	0.00	0.00	0.04	0.00	0.00	0.15	0.00	1.00	0.67	0.00	0.00	0.00	0.00	0.00	0.24
Milwaukee, Wisconsin	0.00	1.00	0.33	0.00	1.00	0.44	0.00	0.00	0.13	0.00	0.00	0.00	0.00	1.00	0.46
Minneapolis, Minnesota	0.00	1.00	0.68	0.00	0.00	0.08	0.00	0.00	0.00	0.00	0.00	0.00	0.00	0.50	0.25

Nashville, Tennessee	0.00	1.00	0.54	0.00	0.00	0.18	0.00	0.00	0.00	0.00	0.00	0.00	0.00	0.00	0.13
New Orleans, Louisiana	0.00	1.00	0.32	0.00	1.00	0.59	0.00	0.00	0.00	0.00	0.00	0.00	0.00	1.00	0.37
New York, New York	0.00	1.00	0.32	0.00	0.00	0.20	0.00	0.00	0.19	0.00	0.00	0.06	0.00	0.00	0.17
Newark, New Jersey	0.00	0.00	0.02	0.00	1.00	0.60	0.00	1.00	0.31	0.00	0.00	0.00	0.00	1.00	0.45
Norfolk, Virginia	0.00	1.00	0.52	0.00	1.00	0.35	0.00	0.00	0.00	0.00	0.00	0.00	0.00	0.00	0.12
North Las Vegas, Nevada	0.00	0.00	0.04	0.00	0.00	0.02	0.00	1.00	0.28	0.00	0.00	0.00	0.00	0.00	0.13
Oakland, California	0.00	0.00	0.22	0.00	0.00	0.04	0.00	0.00	0.17	0.00	0.00	0.03	0.00	0.00	0.12
Oklahoma, Oklahoma	0.00	1.00	0.57	0.00	0.00	0.10	0.00	0.00	0.13	0.00	0.00	0.00	0.00	0.00	0.23
Omaha, Nebraska	0.00	1.00	0.72	0.00	0.00	0.08	0.00	0.00	0.09	0.00	0.00	0.00	0.00	0.00	0.16
Orlando, Florida	0.00	0.50	0.25	0.00	0.00	0.22	0.00	0.00	0.24	0.00	0.00	0.00	0.00	0.00	0.16
Philadelphia, Pennsylvania	0.00	1.00	0.36	0.00	1.00	0.40	0.00	0.00	0.06	0.00	0.00	0.01	0.00	1.00	0.36
Phoenix, Arizona	0.00	1.00	0.49	0.00	0.00	0.00	0.00	1.00	0.35	0.00	0.00	0.00	0.00	0.00	0.24
Pittsburgh, Pennsylvania	0.00	1.00	0.71	0.00	0.00	0.21	0.00	0.00	0.00	0.00	0.00	0.00	0.00	1.00	0.28
Plano, Texas	0.00	1.00	0.65	0.00	0.00	0.00	0.00	0.00	0.05	0.00	0.00	0.05	0.00	0.00	0.00
Raleigh, North Carolina	0.00	1.00	0.72	0.00	0.00	0.15	0.00	0.00	0.00	0.00	0.00	0.00	0.00	0.00	0.15
Reno, Nevada	0.00	1.00	0.73	0.00	0.00	0.00	0.00	0.00	0.12	0.00	0.00	0.00	0.00	0.00	0.10
Richmond, Virginia	0.00	1.00	0.42	0.00	1.00	0.45	0.00	0.00	0.00	0.00	0.00	0.00	0.00	1.00	0.32
Riverside, California	0.00	0.00	0.14	0.00	0.00	0.00	0.00	1.00	0.59	0.00	0.00	0.00	0.00	0.00	0.14
Sacramento, California	0.00	1.00	0.26	0.00	0.00	0.00	0.00	0.00	0.09	0.00	0.00	0.00	0.00	0.00	0.21
San Antonio, Texas	0.00	0.00	0.11	0.00	0.00	0.00	0.00	1.00	0.69	0.00	0.00	0.00	0.00	0.00	0.22
San Bernardino, California	0.00	0.00	0.00	0.00	0.00	0.00	1.00	1.00	0.82	0.00	0.00	0.00	0.00	1.00	0.46
San Diego, California	0.00	1.00	0.49	0.00	0.00	0.00	0.00	0.00	0.21	0.00	0.00	0.04	0.00	0.00	0.09
San Francisco, California	0.00	1.00	0.39	0.00	0.00	0.01	0.00	0.00	0.01	0.00	0.00	0.19	0.00	0.00	0.06
San Jose, California	0.00	0.00	0.16	0.00	0.00	0.00	0.00	0.00	0.21	0.00	0.00	0.23	0.00	0.00	0.01
Santa Ana, California	0.00	0.00	0.00	0.00	0.00	0.00	1.00	1.00	0.87	0.00	0.00	0.04	0.00	0.00	0.09
Seattle, Washington	1.00	1.00	0.80	0.00	0.00	0.00	0.00	0.00	0.00	0.00	0.00	0.03	0.00	0.00	0.08
St. Louis, Missouri	0.00	1.00	0.40	0.00	1.00	0.49	0.00	0.00	0.00	0.00	0.00	0.00	0.00	1.00	0.38

St. Paul, Minnesota	0.00	1.00	0.52	0.00	0.00	0.04	0.00	0.00	0.00	0.00	0.00	0.01	0.00	0.00	0.17
St. Petersburg, Florida	0.75	1.00	0.75	0.00	0.00	0.19	0.00	0.00	0.00	0.00	0.00	0.00	0.00	0.00	0.07
Stockton, California	0.00	0.00	0.07	0.00	0.00	0.00	0.00	1.00	0.28	0.00	0.00	0.00	0.00	1.00	0.32
Tampa, Florida	0.00	1.00	0.43	0.00	0.00	0.18	0.00	0.00	0.12	0.00	0.00	0.00	0.00	1.00	0.34
Toledo, Ohio	0.00	1.00	0.57	0.00	1.00	0.28	0.00	0.00	0.00	0.00	0.00	0.00	0.00	1.00	0.41
Tucson, Arizona	0.00	1.00	0.55	0.00	0.00	0.00	0.00	1.00	0.31	0.00	0.00	0.00	0.00	1.00	0.31
Tulsa, Oklahoma	0.00	1.00	0.58	0.00	0.00	0.09	0.00	0.00	0.02	0.00	0.00	0.00	0.00	0.00	0.21
Virginia Beach, Virginia	0.00	1.00	0.73	0.00	0.00	0.03	0.00	0.00	0.00	0.00	0.00	0.00	0.00	0.00	0.01
Washington, District of Columbia	0.00	1.00	0.35	0.00	1.00	0.47	0.00	0.00	0.00	0.00	0.00	0.00	0.00	0.00	0.16
Wichita, Kansas	0.25	1.00	0.74	0.00	0.00	0.05	0.00	0.00	0.03	0.00	0.00	0.00	0.00	0.00	0.21
Winston-Salem, North Carolina	0.00	1.00	0.49	0.00	1.00	0.26	0.00	0.00	0.00	0.00	0.00	0.00	0.00	1.00	0.42

Table S3. Descriptive statistics by city (cont.)

	Total trips			PM2.5 - Residential			PM2.5 - Adjacent			PM2.5 -Network (non-adjacent)		
**City**	**25th**	**75th**	**Mean**	**25th**	**75th**	**Mean**	**25th**	**75th**	**Mean**	**25th**	**75th**	**Mean**
Albuquerque, New Mexico	31917	61790	48570	6.40	6.76	6.57	6.37	6.74	6.54	6.53	6.62	6.57
Anaheim, California	75069	127052	99305	11.82	12.15	12.02	11.81	12.15	12.01	11.69	11.80	11.74
Arlington, Texas	75458	139052	118216	8.74	8.80	8.77	8.74	8.80	8.77	8.73	8.77	8.75
Atlanta, Georgia	34165	85004	62009	10.73	10.81	10.76	10.73	10.81	10.76	10.65	10.69	10.67
Aurora, Colorado	50462	77832	68771	7.34	7.89	7.60	7.35	7.86	7.59	7.33	7.65	7.48
Austin, Texas	39952	88527	69978	8.16	8.24	8.19	8.15	8.24	8.19	8.15	8.19	8.17
Bakersfield, California	46991	76328	81622	12.55	13.02	12.72	12.52	12.95	12.67	12.65	12.76	12.71
Baltimore, Maryland	22981	43263	34729	8.50	8.61	8.55	8.50	8.61	8.55	8.49	8.55	8.52
Baton Rouge, Louisiana	30804	75132	56103	10.16	10.24	10.18	10.14	10.23	10.17	10.09	10.13	10.11
Boston, Massachusetts	27867	53570	41948	6.68	6.73	6.70	6.68	6.73	6.69	6.63	6.66	6.64
Buffalo, New York	22974	42920	33964	7.73	7.83	7.77	7.72	7.83	7.77	7.72	7.76	7.72
Chandler, Arizona	60138	117348	89910	8.22	8.44	8.33	8.21	8.43	8.32	8.07	8.13	8.10
Charlotte, North Carolina	43920	90589	71122	9.52	9.70	9.61	9.53	9.70	9.60	9.56	9.64	9.60
Chesapeake, Virginia	57519	128487	96295	7.12	7.30	7.22	7.12	7.29	7.21	7.19	7.24	7.22
Chicago, Illinois	27253	62211	47328	9.73	9.91	9.81	9.73	9.91	9.81	9.72	9.83	9.78
Chula Vista, California	58351	88436	107262	9.09	9.16	9.12	9.09	9.15	9.11	9.07	9.09	9.08
Cincinnati, Ohio	18796	46555	34271	9.40	9.56	9.48	9.41	9.55	9.48	9.46	9.53	9.49
Cleveland, Ohio	11729	26902	19998	9.57	9.71	9.63	9.57	9.71	9.63	9.52	9.60	9.56
Colorado Springs, Colorado	39981	81166	66187	5.39	5.56	5.47	5.37	5.56	5.45	5.41	5.46	5.44
Columbus, Ohio	28944	65390	51253	8.76	8.85	8.79	8.76	8.85	8.79	8.74	8.79	8.76
Corpus Christi, Texas	46848	93782	74973	7.45	7.63	7.53	7.46	7.63	7.53	7.51	7.55	7.53
Dallas, Texas	48518	90076	73899	8.79	8.88	8.83	8.79	8.88	8.83	8.80	8.86	8.83
Denver, Colorado	34437	62122	52082	7.48	7.94	7.68	7.46	7.93	7.67	7.37	7.64	7.50

Des Moines, Iowa	30600	54340	46436	7.58	7.62	7.60	7.58	7.62	7.60	7.58	7.59	7.58
Detroit, Michigan	12591	29684	22997	9.59	9.79	9.69	9.59	9.78	9.68	9.57	9.65	9.61
Durham, North Carolina	26821	59498	48013	9.24	9.37	9.30	9.24	9.37	9.29	9.24	9.27	9.26
Fort Wayne, Indiana	25705	61219	42944	9.01	9.03	9.02	9.01	9.03	9.02	9.01	9.02	9.02
Fort Worth, Texas	53132	119307	117243	8.49	8.65	8.57	8.49	8.65	8.57	8.55	8.66	8.61
Fremont, California	40165	75589	56918	8.18	8.26	8.22	8.17	8.26	8.22	8.13	8.16	8.14
Fresno, California	35383	63288	55826	12.61	13.24	12.86	12.62	13.22	12.85	12.59	12.80	12.68
Garland, Texas	75358	123134	99454	8.78	8.85	8.81	8.78	8.84	8.81	8.80	8.84	8.82
Glendale, Arizona	53738	84223	71226	7.24	8.02	7.65	7.24	7.99	7.65	7.44	7.86	7.65
Greensboro, North Carolina	31369	63734	49170	8.74	8.84	8.79	8.75	8.84	8.79	8.79	8.82	8.80
Houston, Texas	50885	94037	79133	9.88	10.08	9.92	9.87	10.08	9.91	9.81	9.95	9.84
Indianapolis (balance), Indiana	31753	67119	56861	9.68	9.82	9.74	9.67	9.82	9.74	9.64	9.71	9.67
Irvine, California	64806	162494	135985	10.57	10.99	10.81	10.55	10.99	10.80	10.91	11.02	10.93
Irving, Texas	58232	90815	74155	8.86	8.89	8.87	8.86	8.88	8.87	8.84	8.85	8.85
Jacksonville, Florida	52134	127344	101995	8.10	8.36	8.21	8.11	8.34	8.20	8.09	8.21	8.14
Jersey, New Jersey	35865	70565	55532	8.88	8.94	8.91	8.88	8.94	8.91	8.73	8.80	8.77
Kansas, Missouri	27341	59274	53898	7.67	7.91	7.79	7.66	7.91	7.79	7.67	7.79	7.73
Las Vegas, Nevada	48592	82395	69982	5.35	6.95	6.09	5.31	6.93	6.08	6.17	6.81	6.51
Long Beach, California	51742	78352	65854	10.99	11.67	11.34	10.98	11.69	11.34	11.34	11.50	11.45
Los Angeles, California	41852	73090	58729	10.49	11.96	11.24	10.49	11.96	11.24	10.81	11.74	11.27
Louisville, Kentucky	55389	109353	84679	9.02	9.15	9.08	9.01	9.15	9.07	9.11	9.14	9.12
Lubbock, Texas	46045	99600	83566	6.57	6.63	6.59	6.55	6.63	6.59	6.57	6.58	6.58
Memphis, Tennessee	24118	69078	50220	9.02	9.16	9.08	9.02	9.16	9.08	9.00	9.07	9.04
Mesa, Arizona	45474	83777	70831	7.38	7.70	7.53	7.38	7.70	7.53	7.63	7.79	7.72
Miami, Florida	52933	100583	80630	7.49	7.67	7.58	7.49	7.68	7.58	7.52	7.63	7.58
Milwaukee, Wisconsin	20009	36881	29937	8.89	8.97	8.93	8.89	8.97	8.93	8.92	8.95	8.93
Minneapolis, Minnesota	26831	46671	38131	7.42	7.48	7.45	7.41	7.48	7.44	7.34	7.38	7.36

Nashville, Tennessee	39018	75416	61984	9.15	9.46	9.27	9.14	9.45	9.26	9.19	9.31	9.24
New Orleans, Louisiana	15604	34454	28934	8.04	8.15	8.08	8.03	8.15	8.08	8.10	8.13	8.11
New York, New York	37155	77683	61759	8.24	8.61	8.40	8.24	8.61	8.40	8.36	8.56	8.43
Newark, New Jersey	35586	62289	51428	8.89	8.95	8.92	8.89	8.95	8.92	8.76	8.80	8.78
Norfolk, Virginia	27201	47794	37967	7.30	7.37	7.33	7.30	7.37	7.33	7.25	7.28	7.26
North Las Vegas, Nevada	51519	107483	86449	5.77	6.74	6.23	5.76	6.71	6.22	6.32	6.76	6.53
Oakland, California	25628	49530	39273	8.10	8.32	8.19	8.10	8.31	8.19	8.11	8.14	8.13
Oklahoma, Oklahoma	26476	85936	69249	8.28	8.38	8.32	8.27	8.38	8.31	8.28	8.32	8.30
Omaha, Nebraska	31529	60811	49404	7.98	8.05	8.01	7.97	8.04	8.00	7.98	8.01	7.99
Orlando, Florida	57061	127028	109735	7.74	7.90	7.82	7.73	7.90	7.81	7.73	7.81	7.77
Philadelphia, Pennsylvania	33918	70195	53836	9.83	9.94	9.88	9.83	9.94	9.88	9.81	9.90	9.86
Phoenix, Arizona	45541	80871	66811	7.19	8.46	7.85	7.19	8.45	7.84	7.38	8.10	7.77
Pittsburgh, Pennsylvania	15993	31500	24873	10.39	10.56	10.47	10.39	10.56	10.47	10.33	10.44	10.38
Plano, Texas	87535	148013	118266	8.90	8.94	8.92	8.90	8.93	8.92	8.88	8.90	8.89
Raleigh, North Carolina	54873	118888	95623	9.40	9.50	9.44	9.39	9.49	9.43	9.39	9.43	9.41
Reno, Nevada	38164	63215	55696	6.64	6.74	6.69	6.63	6.73	6.68	6.68	6.70	6.69
Richmond, Virginia	20257	49392	36063	8.19	8.26	8.22	8.19	8.26	8.22	8.17	8.19	8.18
Riverside, California	43781	76056	64779	12.52	12.89	12.69	12.49	12.90	12.68	12.18	12.44	12.31
Sacramento, California	34064	74794	56629	8.84	9.01	8.91	8.82	9.00	8.90	8.69	8.86	8.77
San Antonio, Texas	57681	113699	92287	8.19	8.35	8.26	8.19	8.35	8.25	8.22	8.28	8.25
San Bernardino, California	48495	73573	62129	10.63	11.33	10.99	10.62	11.32	10.95	11.24	11.44	11.36
San Diego, California	46412	82272	68448	9.01	9.25	9.11	9.00	9.25	9.10	9.02	9.11	9.06
San Francisco, California	30743	61579	49507	7.57	7.90	7.73	7.56	7.90	7.73	7.69	7.83	7.76
San Jose, California	45597	68729	59467	7.67	8.12	7.87	7.67	8.10	7.87	7.76	7.95	7.86
Santa Ana, California	60320	87159	74600	11.17	11.43	11.29	11.16	11.40	11.29	11.20	11.33	11.26
Seattle, Washington	36017	64111	50944	6.25	6.49	6.37	6.26	6.49	6.37	6.29	6.41	6.35
St. Louis, Missouri	22449	43930	34377	9.23	9.34	9.28	9.23	9.34	9.28	9.20	9.27	9.23

St. Paul, Minnesota	30236	60016	44003	7.44	7.48	7.46	7.44	7.48	7.46	7.37	7.39	7.38
St. Petersburg, Florida	44342	71718	60864	7.64	7.92	7.78	7.65	7.92	7.78	7.78	7.87	7.83
Stockton, California	30785	48944	48373	10.86	11.15	11.00	10.83	11.15	10.97	10.86	10.92	10.89
Tampa, Florida	38192	82863	66653	8.40	8.48	8.41	8.40	8.48	8.41	8.31	8.35	8.31
Toledo, Ohio	17524	44387	31905	9.11	9.16	9.13	9.11	9.15	9.13	9.10	9.12	9.11
Tucson, Arizona	32993	57501	47102	6.33	6.66	6.46	6.31	6.62	6.45	6.41	6.51	6.46
Tulsa, Oklahoma	27775	62446	51331	9.47	9.57	9.52	9.47	9.56	9.51	9.49	9.51	9.50
Virginia Beach, Virginia	52252	96130	76009	6.95	7.16	7.04	6.95	7.16	7.05	7.07	7.14	7.11
Washington, District of Columbia	44015	78524	62817	8.79	8.88	8.82	8.79	8.87	8.82	8.72	8.77	8.74
Wichita, Kansas	32157	60852	54151	7.75	7.80	7.77	7.74	7.81	7.76	7.74	7.75	7.74
Winston-Salem, North Carolina	22280	59811	44259	8.49	8.62	8.55	8.48	8.62	8.55	8.54	8.57	8.55

Table S4. Regression adjusted $PM_{2.5}$ in the neighborhoods that residents travel to weighted by the number of trips

	Estimate	SE	95% CI	
**Group**			**Lower**	**Upper**
White	7.81	0.004	7.81	7.82
Asian	8.71	0.013	8.69	8.74
Black	8.71	0.005	8.70	8.72
Hispanic	8.78	0.005	8.77	8.79
Non Poor	8.19	0.003	8.19	8.20
Poor	8.75	0.005	8.74	8.76

Estimated marginal means are from Ordinary Least Squares regressions that include total population and city-fixed effects.

Table S5. Regression adjusted $PM_{2.5}$ in residential neighborhoods (Residential), adjacent neighborhoods (Adjacent) and the non-adjacent neighborhoods that residents travel to (Network) weighted by the number of trips

	**Residential**				**Adjacent**				**Network**			
	**Estimate**	**SE**	**95% CI**		**Estimate**	**SE**	**95% CI**		**Estimate**	**SE**	**95% CI**	
**Group**			**Lower**	**Upper**			**Lower**	**Upper**			**Lower**	**Upper**
White	7.81	0.006	7.80	7.82	7.81	0.007	7.80	7.82	7.79	0.005	7.78	7.80
Asian	8.74	0.020	8.70	8.78	8.74	0.007	8.70	8.78	8.70	0.007	8.68	8.72
Black	8.72	0.007	8.71	8.74	8.72	0.003	8.70	8.73	8.71	0.005	8.70	8.72
Hispanic	8.85	0.007	8.84	8.87	8.85	0.012	8.83	8.86	8.77	0.007	8.76	8.78
Non Poor	8.21	0.006	8.20	8.22	8.21	0.004	8.20	8.22	8.19	0.003	8.18	8.19
Poor	8.80	0.020	8.79	8.81	8.79	0.004	8.78	8.81	8.74	0.004	8.73	8.75

Estimated marginal means are from Ordinary Least Squares regressions that include total population and city-fixed effects.

Table S6. Regression adjusted $PM_{2.5}$ in residential neighborhoods (Residential), adjacent neighborhoods (Adjacent) and the non-adjacent neighborhoods that residents travel to (Network) weighted by the number of trips by race/ethnicity and poverty status

	**Residential**				**Adjacent**				**Network**			
	**Estimate**	**SE**	**95% CI**		**Estimate**	**SE**	**95% CI**		**Estimate**	**SE**	**95% CI**	
**Group**			**Lower**	**Upper**			**Lower**	**Upper**			**Lower**	**Upper**
Non Poor												
White	7.77	0.005	7.76	7.78	7.77	0.005	7.76	7.78	7.79	0.003	7.78	7.80
Asian	8.70	0.021	8.66	8.74	8.69	0.021	8.65	8.73	8.67	0.012	8.65	8.70
Black	8.71	0.009	8.69	8.73	8.70	0.009	8.69	8.72	8.70	0.005	8.69	8.71
Hispanic	8.79	0.008	8.77	8.80	8.78	0.008	8.76	8.80	8.73	0.005	8.72	8.74
Poor												
White	8.26	0.018	8.22	8.29	8.25	0.018	8.22	8.29	8.20	0.011	8.22	8.19
Asian	8.85	0.060	8.73	8.97	8.84	0.060	8.72	8.96	8.76	0.036	8.69	8.83
Black	8.74	0.010	8.72	8.76	8.73	0.010	8.71	8.75	8.72	0.006	8.71	8.73
Hispanic	8.94	0.011	8.92	8.97	8.94	0.011	8.92	8.96	8.83	0.006	8.81	8.84

Estimated marginal means are from Ordinary Least Squares regressions that include total population and city-fixed effects.

Table S7. Within-MSA travel patterns by neighborhood racial/ethnic and poverty type, Proportion of trips

	**Residential**				**Adjacent**				**Network**			
	**Estimate**	**SE**	**95% CI**		**Estimate**	**SE**	**95% CI**		**Estimate**	**SE**	**95% CI**	
**Group**			**Lower**	**Upper**			**Lower**	**Upper**			**Lower**	**Upper**
Non Poor												
White	0.08	0.004	0.07	0.09	0.19	0.007	0.18	0.20	0.73	0.007	0.72	0.74
Asian	0.06	0.016	0.03	0.10	0.17	0.027	0.11	0.22	0.77	0.029	0.71	0.83
Black	0.06	0.007	0.05	0.07	0.14	0.011	0.12	0.16	0.80	0.012	0.78	0.83
Hispanic	0.07	0.007	0.06	0.09	0.17	0.011	0.15	0.19	0.76	0.012	0.73	0.78
Poor												
White	0.13	0.020	0.09	0.17	0.20	0.025	0.16	0.25	0.66	0.028	0.61	0.72
Asian	0.12	0.064	-0.01	0.24	0.23	0.090	0.05	0.40	0.66	0.097	0.47	0.85
Black	0.08	0.008	0.06	0.09	0.14	0.011	0.12	0.17	0.78	0.013	0.75	0.81
Hispanic	0.09	0.010	0.07	0.11	0.17	0.014	0.14	0.20	0.74	0.016	0.71	0.77

Estimated marginal proportions are from logistic regressions that include total population and city-fixed effects.

Table S8. Within-MSA travel patterns by neighborhood racial/ethnic and poverty type, Average distance travelled

Group	Estimate	SE	95% CI Lower	95% CI Upper
Non Poor				
White	-1.08	0.001	-1.08	-1.08
Asian	-1.07	0.004	-1.07	-1.06
Black	-1.06	0.002	-1.06	-1.05
Hispanic	-1.08	0.002	-1.08	-1.08
Poor				
White	-1.11	0.004	-1.12	-1.10
Asian	-1.10	0.012	-1.13	-1.08
Black	-1.08	0.002	-1.09	-1.08
Hispanic	-1.11	0.002	-1.11	-1.11

Estimated marginal means are from Ordinary Least Squares regressions that include total population and city-fixed effects. Distance is within-MSA standardized.
